# Effects of adaptive feedback through a digital tool – a mixed-methods study on the course of self-regulated learning

**DOI:** 10.1007/s10639-024-12510-8

**Published:** 2024-03-02

**Authors:** Mathias Mejeh, Livia Sarbach, Tina Hascher

**Affiliations:** 1 Department of Research in School and Learning, Institute of Educational Science, University of Bern, Fabrikstrasse 8, 3012 Bern, Switzerland; 2 Department of Education Studies, University of California, San Diego, 9625 Scholars Drive North, La Jolla, CA 92093-0070 USA

**Keywords:** Self-regulated learning, Adaptive learning technology, Feedback, Mixed-methods

## Abstract

Lifelong learning is emerging as a key priority for promoting equity and sustainability in societies. Self-regulated learning (SRL) is a fundamental requirement for achieving successful lifelong learning, and digitization is increasingly influential in this regard. This mixed-methods study explores the degree to which adaptive learning technology (ALT) can assist university students in their SRL with timely and personalized support. Additionally, the study examines how students perceive this feedback and incorporate it into their learning behavior. Using hierarchical linear modeling, we investigated the development of SRL over a 9-week period. Semi-structured interviews were conducted with purposively selected learners, based on stimulated recalls. The quantitative results demonstrate positive development in certain components of SRL. Furthermore, the results indicate that metacognitive activity can be partially predicted by motivational and emotional states. The qualitative findings reveal that learners have varying perceptions of feedback received from ALT and integrate it into their learning behaviors based on their individual benefits. The results support the assumption that feedback provided through educational technology must be precisely tailored to the needs of learners, taking into account the dynamics of their individual learning processes. The study contributes to the ongoing discussion on the design of educational technology.

**Supplementary Information:**

The online version contains supplementary material available at 10.1007/s10639-024-12510-8.

## Introduction

Lifelong learning is crucial in the twenty-first century for addressing global challenges such as health, climate crises, and technological transformation (UNESCO, 2022). Self-Regulated Learning (SRL) is essential for lifelong learning, involving independent and self-directed behavior to improve knowledge and skills (Lüftenegger et al., 2012; Wigfield et al., 2011) and is even considered the centerpiece of learning in the twenty-first-century (Anthonysamy et al., 2020). Students who successfully regulate their learning processes show better academic performance (e.g., Richardson et al., 2012) and experience a higher sense of well-being (Davis & Hadwin, 2021).

Contemporary SRL research is increasingly focusing on student learning in digital learning environments (Broadbent et al., 2020a; Kuhnel et al., 2018), which is both prerequisite and a result of the digitization of university teaching. Modern educational technology and online learning environments can be an essential prerequisite for a better understanding of SRL (Winne, 2022). For students in higher education, successful SRL is crucial (Jansen et al., 2019) as they – in contrast to learners in elementary and secondary school – experience far more responsibility and freedom for their learning (Sitzmann & Ely, 2011). While the relevance of SRL in higher education has been demonstrated numerous times, more work is needed to better understand how to foster and empower students in terms of their SRL through adaptive learning technologies (Broadbent & Poon, 2015). Several aspects need more attention: Firstly, the successful regulation of one’s learning process is dynamic, temporal, and adaptive (Greene et al., 2021). Accurately capturing and analyzing SRL remains a complex task for researchers (Molenaar, 2014; Winne & Perry, 2000). Secondly, higher education is characterized by an increasingly diverse student body in which clear differences in the effective use of SRL strategies are becoming apparent (de Bruijn-Smolders et al., 2016; Dörrenbächer-Ulrich et al., 2021; Jansen et al., 2019; Peverly et al., 2003; Wolters & Brady, 2021). However, personalized, real-time feedback that best assists learners in their SRL is still rarely used in higher education (Tsai et al., 2020). Third, students not only need ongoing feedback on their learning process but also need to find themselves in a learning environment that is conducive to SRL (Dignath & Veenman, 2021).

This study aims to contribute to an understanding of the SLR process by using a digital tool called “studybuddy” to capture and analyze task-related SRL and examine the impact of adaptive feedback on students’ SRL. The study combines qualitative and quantitative methods to gain a deeper understanding of SRL development and promotion (Anthonysamy et al., 2020; Kelle, 2015; McCardle & Hadwin, 2015; Pekrun, 2020).

## Theoretical background

### Self-regulated learning: Processes & strategies

SRL is defined as independent, self-directed behavior by individuals seeking to improve their knowledge and skills (Paris & Paris, 2001). In this context, learning takes place under intentional regulation and monitoring of cognition, emotion, behavior, and motivation (Pintrich, 2004; Winne & Hadwin, 1998; Zimmerman, 2008). Through the intentional use of (meta-) cognitive, motivational, and emotional strategies, students gain a deeper understanding of their own learning as they try to achieve previously set learning goals (Boekaerts, 2011). SRL, in the early stages of its theoretical conceptualization, was primarily defined and measured as a dispositional trait (Winne & Perry, 2000). Contemporary views see SRL as a dynamic and iterative process in which different components of learning (viewed as states) unfold over time (Greene et al., 2021; Molenaar & Järvelä, 2014). In research focused on the dynamics of SRL, time units are conceptualized differently, resulting in artificial divisions. These divisions may encompass a range of temporal segments, including individual lessons or complete teaching units spanning several weeks. It is therefore fundamental to link the segmentation of defined periods to clear guidelines and justify them theoretically (Molenaar, 2014). Moreover, regulatory strategies are often directly included in the definitional framework of SRL because they play a fundamental role in each stage of the learning process (Matcha et al., 2019) and their use is associated with the active construction of knowledge (Winne, 2005; Zimmerman, 2008).

**Fig. 1:**
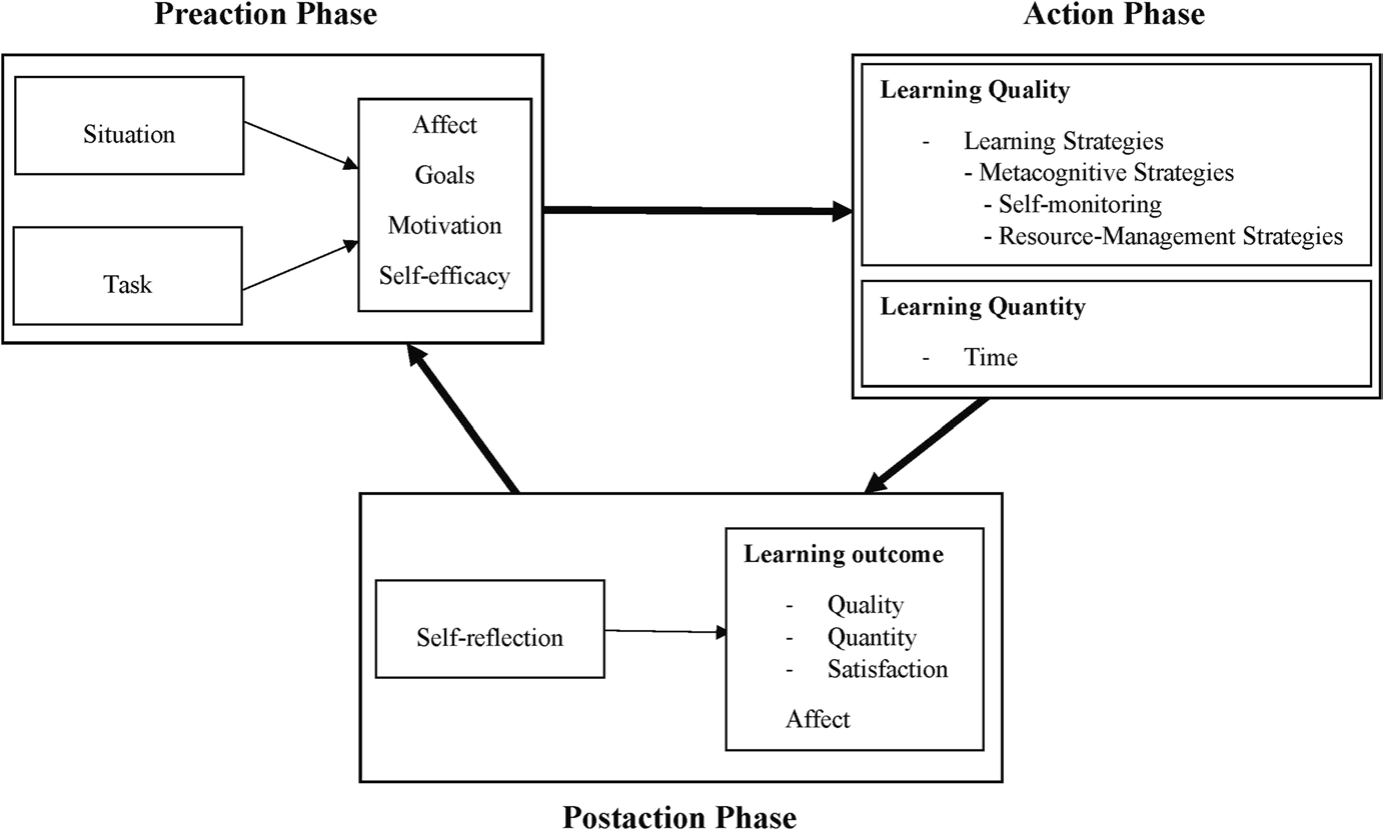
Self-regulation process model of learning (Schmitz & Wiese, 2006)

In this paper, the self-regulation process model (Schmitz, 2001; Schmitz & Wiese, 2006) provides the theoretical basis, as it focuses on the learning phase-related application of self-regulation strategies (see Fig. 1). The model is based on Zimmerman’s (2000) cyclical phase model, whose central importance is the division of learning into a pre-actional, actional, and post-actional phase (Schmitz, 2001). Accordingly, learning is divided into different episodes (e.g., a task, a lesson, or a whole school day) that proceed cyclically, whereby – due to a feedback loop – preceding phases influence subsequent phases (Bellhäuser et al., 2022). The self-regulation process model emphasizes the dynamic course of SRL and the role of feedback.

During the pre-actional phase of the self-regulation process model of learning, various motivational and emotional factors are relevant for the success of SRL. At the beginning of the learning cycle, learners are confronted with a task, which can be either self-set or externally-set, highlighting the importance of task value (Eccles, 1983; Pintrich, 1999). To tackle a task, learners autonomously set goals, while goal orientation is considered significant as predictor for metacognitive activity (Pintrich, 2004; Wigfield et al., 2011). Additionally, self-efficacy is identified as a key factor for successful SRL in the pre-actional stage, as it plays a crucial role in planning learners’ strategy use (Bandura, 1986; Pintrich et al., 1991). Furthermore, the emotional state significantly impacts this learning phase as emotions can influence motivation, cognition and learning intentions (Boekaerts, 2011; Efklides, 2011). Once the task analysis process is finished, learners develop plans on how to achieve their set goals.

The actional phase and the actual processing of the task begin after the planning is completed. Schmitz and Wiese (2006) draw a fundamental distinction between the quantity and the quality of learning activities: While the quantity of learning basically addresses how much time is invested in learning, the quality aims at the question of how learners learn. Of central importance with regard to the quality of learning is the application of metacognitive (e.g., monitoring) as well as cognitive (e.g., organization) strategies (Pintrich et al., 1991; Winne & Hadwin, 1998). In addition to cognitive components, emotional as well as motivational states are of importance. For example, a purposeful application of volitional strategies and resource management is indispensable in this phase for learners in order to effectively deal with distractions, to engage in learning or to avoid procrastinating behavior (Pintrich, 1999).

The learning episode concludes with the post-actional phase, in which the learning and task results are reflected and evaluated accordingly. For this purpose, the goals set at the beginning of the learning process are compared with the goals achieved. Learners assess their satisfaction with regard to the quantity of what they have learned (quantitative learning aspect) and their understanding (qualitative learning aspect). If the achieved result is not satisfactory, learners with strong SRL skills take action to optimize their learning process in the next learning episode. This happens, for example, by adjusting learning strategies or setting more realistic goals. If the learning outcome is satisfactory, the chosen strategies are maintained (Schmitz, 2001). Satisfaction and dissatisfaction, respectively, are closely linked to positive and negative emotions, highlighting the significance of emotional states in metacognitive regulation during the post-actional phase (Boekaerts, 1997; Pekrun et al., 2002; Schmitz & Wiese, 2006).

According to Schmitz’s process model, effective strategy application is central to successful SRL, and continuous monitoring and adaptation of strategies, as suggested by Zimmerman (2008), are necessary prerequisites for achieving this success. SRL strategies positively influence academic performance, enabling learners to acquire the subject matter in a structured and methodological way (Broadbent, 2017; Broadbent & Poon, 2015; Richardson et al., 2012). Furthermore, monitoring one’s learning process, emotion regulation and motivational incentives are also significant for learning (Streblow & Schiefele, 2006). Strategies play a fundamental role in each stage of the learning process since learners can optimize the learning process by using appropriate learning strategies, allowing them to learn in a self-regulated way (Matcha et al., 2019). The type and frequency of use of SRL strategies vary (Dörrenbächer & Perels, 2016), which can be attributed to different experiences with strategies, divergent preferences, or different frequency of use for them (Broadbent et al., 2020b; Dörrenbächer & Perels, 2016). Theobald and Bellhäuser (2022) distinguish between learners who lack knowledge of effective strategies, and those who know effective strategies but have difficulty applying them in different contexts. They emphasize the significance of accounting for the dynamic nature of SRL when developing interventions, as students may necessitate varying forms of support based on situational factors. The deployment of effective strategies is closely associated with adaptive feedback (Matcha et al., 2019).

### The role of feedback and adaptive learning technologies in enhancing self-regulated learning

Feedback plays a crucial role in enhancing learning outcomes in higher education, with particular emphasis on its potential to improve students’ SRL (e.g., Cornelius-White, 2007; Shute, 2008). Feedback is defined as the process by which learners extract information from various sources (e.g., teachers, fellow students, friends, family members, or automated computer-based systems) and modify their learning and work processes accordingly (Boud & Molloy, 2013; Carless, 2015). Feedback can be classified as internal or external (Winne & Hadwin, 1998), where external feedback refers to those regulations that support learners in monitoring their learning process by providing them with information about their progress. External feedback informs learners about gaps between their current performance and their desired objectives and helps to regulate their learning accordingly to reduce these discrepancies (Devolder et al., 2012; Hattie & Timperley, 2007). During this regulatory process, learners can generate internal feedback (Nicol & Macfarlane‐Dick, 2006). Feedback is therefore often considered a dialogic process in which learners are expected to transform external feedback into internal feedback and utilize it effectively (Carless & Winstone, 2020). The link between the two types of feedback and SRL becomes evident during the monitoring of tactics, strategies and the discrepancy between goals and outcomes (Butler & Winne, 1995). The internal feedback generated by this metacognitive process assists learners in regulating their knowledge, beliefs, goals, tactics, and strategies. Learners can decide to modify their strategies to achieve their goals (Hattie & Timperley, 2007). External feedback can assist learners in adapting, selecting as well as utilizing regulatory strategies, thereby stimulating internal feedback processes and enhancing SRL skills (Lim et al., 2021). Learners with higher SRL skills can more effectively self-generate internal feedback compared to learners with lower SRL skills since they can make appropriate regulations and possess adequate knowledge about potential learning strategies or resource utilization (Chou & Zou, 2020; Matcha et al., 2019).

While the significance of rich and tailored feedback has long been acknowledged, its scalability in modern educational settings has been limited (Matcha et al., 2019). One promising solution for personalized and timely feedback processes is adaptive learning technology (ALT) (Martin et al., 2020; Xie et al., 2019). ALTs can trigger an iterative cycle of self-monitoring (internal feedback), performance, assessment, and external feedback based on data about learners and their learning environment, thereby providing feedback to learners and instructors to support the assessment and adaptation of the learning process (Schmid et al., 2022a, 2022b). ALTs have been shown to have a significant impact on successful SRL (e.g., Aleven et al., 2017; Molenaar & Van Campen, 2016), with Molenaar and colleagues (Molenaar et al., 2021) suggesting that ALTs take over part of the control and monitoring loop in these technologies, as evidenced by the COPES Model (Winne & Hadwin, 1998).

Moreover, recent work proposes SRL models to describe the complex mediation processes involved in student technology-based learning (Azevedo & Gašević, 2019; Winne & Azevedo, 2014). ALTs have also been used to identify students’ weaknesses in SRL and improve them through the use of clear goals, as demonstrated by Forsyth et al. (2016). They employed an Adaptive Grading Learning System that allowed learners to evaluate their learning and receive timely, personalized feedback from instructors. To date, several ALTs have been developed and implemented with the goal of providing feedback to learners (and instructors) and promoting SRL (e.g., Molenaar et al., 2020; Pammer-Schindler et al., 2018). A literature review conducted by Jivet et al. (2017) examined 26 tools designed to support the learning process in online learning environments, of which 13 were intended to promote SRL. The results demonstrated that SRL can be promoted by such tools if they support learners’ awareness of their learning and reflection on the learning process. The tools suggest learning objectives or learning activities, learning paths, or strategies and tips for SRL (Pammer-Schindler et al., 2018). Thus, ALTs have the potential to provide personalized and scalable feedback to learners, allowing them to monitor and regulate their learning process, ultimately promoting the development of SRL skills.

## “Studybuddy” – a digital tool to foster self-regulated learning

**Fig. 2:**
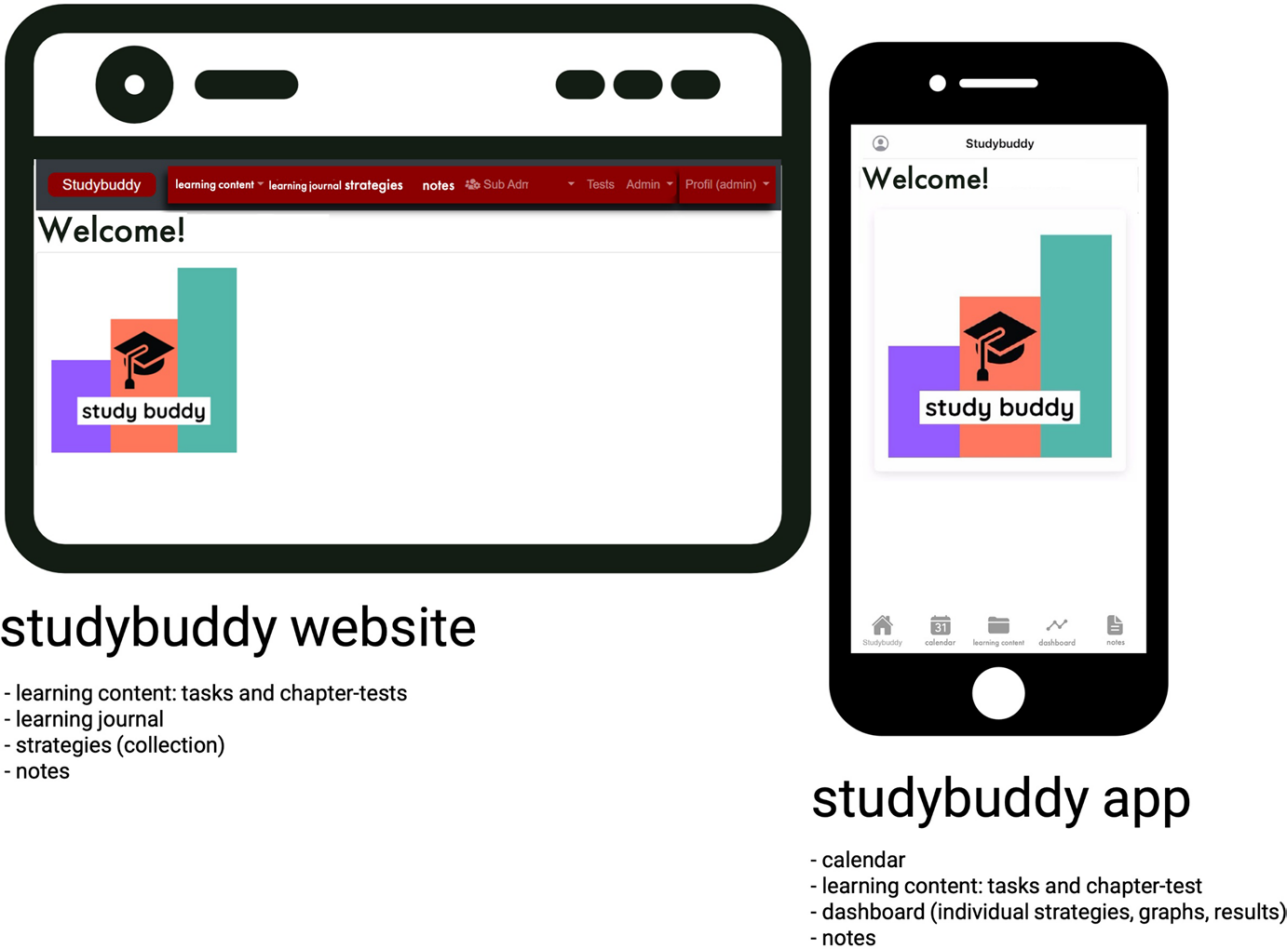
Overview different functions of studybuddy

“Studybuddy” is an ALT in form of a website as well as a corresponding app. The basic idea of studybuddy is a digital tool that fosters SRL and allows to create a learning environment conducive to SRL. In this context, studybuddy provides feedback to learners and teachers. Learning analytics (LA) play a key role in the design and implementation of studybuddy as an ALT, as they enable the platform to provide data-driven insights and personalized support. Studybuddy provides learners with SRL strategies, course tasks, and reminders to complete them. In doing so, learners are provided with feedback through various notes and strategies in the form of prompts. In this way, they receive direct feedback on their learning process. It can also be used as a planning tool, featuring a calendar and a note function. A central feature is that studybuddy supports instructors in monitoring and diagnosing the learning progress of students and responding accordingly. For example, more differentiated feedback can be given (formative or summative), learning content can be structured better, and the teaching can be adapted based on the students’ learning progress. Higher SRL can be achieved by studybuddy interacting directly with the learners concerning their learning processes. A questionnaire-based approach is used to collect SRL-relevant data from learners over time, including information on motivation, emotion, cognition, metacognition, and resource management. The questionnaire is integrated into the digital learning environment as a weekly short survey, administered before and after learners’ complete course tasks. For the most part, the content and functionality of the website is the same as that of the app. All learning materials and the learning strategy collection can be accessed via both media. The app proves to be essential in that its use allows for direct interaction with the users by sending different prompts. The app is also used to display individual learning progress (dashboard function). Studybuddy consists of four parts: an automated feedback system, a digital dashboard, personalized strategies, and a planning tool (Fig. 2).

### Automated feedback system integrated in studybuddy

An automated feedback system provides learners with timely and personalized feedback in the form of push notifications. This feedback is essential for SRL, as it helps learners to adjust their learning strategies and monitor their progress (Nicol & Macfarlane-Dick, 2006). The feedback is pre-programmed and can be send to learners at a predefined time (e.g., push notification of the start of the week) or activated individually based on their needs and preferences (e.g., push notification of new learning strategies). This has been shown to be particularly effective for SRL, as it enables learners to make timely adjustments to their learning strategies and monitor their progress (Dabbagh & Kitsantas, 2012). Shute (2008) found that learners who received immediate and specific feedback through an ALT made significant gains in their performance compared to learners who received delayed or general feedback.

The system can remind students of newly unlocked tasks, keeping them engaged and motivated (Seiler et al., 2018). Notifications are also activated based on certain behavioral patterns within the learning environment, which helps learners to reflect on their learning behaviors and improve their self-regulation skills (Bodily & Verbert, 2017). For instance, when a learner clicks on the “Task completed” button, a notice appears utilizing images and text material to draw their attention to the successful completion of the tasks (Ifenthaler et al., 2021). Additionally, notes become visible in the learning environment at predefined points in time, drawing learners’ attention to the strategy recommendations or strategy collection, which can enhance their learning outcomes (Garcia et al., 2018).

### Digital dashboard integrated in studybuddy

Data collected through the self-reporting process can also be visualized in the form of a digital dashboard, which is an essential feature of LA feedback interventions for promoting SRL (e.g., Ali et al., 2012). Visualizing information is crucial for learners, as it enables them to gain insights into their learning activities and the associated impact (Bodily & Verbert, 2017; Ifenthaler et al., 2021). Digital dashboards have received significant attention in recent years as a tool for visualizing data in educational contexts (Verbert et al., 2013). Studybuddy features a digital dashboard that provides learners real-time access to their learning progress, allowing them to track their learning-related data over a specific time period. This includes diagrams like progress curves, which aid learners in reflecting on their learning and making informed decisions about their next steps (Ifenthaler & Drachsler, 2018). The studybuddy dashboard provides an overview of learning-related data for motivation, emotion, cognition, metacognition and resource management. The average value of these variables is calculated automatically for each short survey and displays the results in the form of progress curves for each individual area, providing a visual representation of the learner’s progress. This feedback intervention empowers learners to take control of and direct their own learning (Nicol & Macfarlane-Dick, 2006), which can ultimately lead to improved learning outcomes.

### Personalized strategies integrated in studybuddy

LA can help to provide insights into how to personalize feedback and interventions to meet the unique needs of the learners (Baker & Inventado, 2014; Kovanović et al., 2018). This allows for individualized LA feedback to be sent to learners in the form of personalized strategy recommendations based on individual motivation, emotion, cognition, metacognition, and resource management data (Fig. 6). The integration of personalized strategy recommendations into a digital learning environment, such as studybuddy, can help to facilitate learners’ adoption and use of these strategies, as well as provide a convenient and accessible resource for their ongoing development (e.g., Dabbagh & Kitsantas, 2012; Järvelä & Hadwin, 2013).

The majority of the strategies used by studybuddy are based on the German version (Metzger, 2017) of the Learning and Study Strategies Inventory (LASSI) (Weinstein et al., 1988). The individual strategies are adjusted and displayed after each collection of learner-related data, both before and after solving tasks. The format takes on the form of short videos that use pictographs and text to clearly illustrate the recommended actions (for an overview of the specific strategies see Appendix A). For example, for motivation, one strategy is to create a sense of achievement. This involves rewarding oneself to an appropriate degree for achieving intermediate goals or embedding an unpleasant task between the completion of two pleasant tasks. In the context of emotion, learners can engage in “expressive writing”, where they write down all their emotions without worrying about grammar or spelling. This activity allows them to reflect on their emotions, resulting in greater emotional regulation and control. For cognition, learners can use the “slow down” strategy, which involves going through the learning material again and taking small breaks to improve understanding and retention. In terms of metacognition, learners can manage their tasks more effectively by using the “task management” strategy, which involves diversifying their work schedule and packing unpleasant tasks between two more pleasant ones. This strategy helps learners prioritize their time, resulting in greater productivity and reduced stress. Finally, when considering resource management, learners can optimize their workspace using the “flow place” strategy, which involves identifying the best place to focus and making sure they are in that environment. By optimizing their resources, learners can better concentrate on their learning tasks. The individual strategies are adjusted and displayed after each collection of learner-related data (before and after solving the tasks, respectively).

### Planning tool integrated in studybuddy

Along with LA feedback, studybuddy has other functionalities that aim to promote different components of SRL and contribute to the design of a learning environment conducive to SRL. For example, a course could be completely configured through the tool. Learners can access all learning content (course content, etc.) directly via the app and website. Research has shown that a well-designed digital learning environment can enhance learners’ SRL skills and academic performance (e.g., Järvelä & Hadwin, 2013). By providing a planning tool within the app, studybuddy supports learners in this important aspect of SRL. Learners can use the tool to set goals, schedule their learning activities, and track their progress over time. This type of tool has been shown to be effective in improving learners’ self-regulation and academic performance (Dabbagh & Kitsantas, 2012). In addition to the planning tool, the note function in studybuddy can also be a useful tool for learners to organize their thoughts and ideas. Research has shown that taking notes can improve learners’ comprehension and retention of information, as well as facilitate metacognitive processes such as reflection and self-evaluation (Kiewra et al., 1991). By providing a digital note-taking function, studybuddy enables learners to capture and organize their thoughts in a convenient and accessible way.

## The present study

Broadbent and Poon (2015) argue that although the importance of SRL has been established in higher education, further research is necessary to enhance our understanding of how to promote and enable students’ SRL. Adaptive feedback is critical to the success of SRL as students frequently struggle with selecting and modifying their strategies to meet the demands of their coursework. This underscores the significance of ALTs for learners, as students should not be perceived as homogeneous groups. To reveal the impact of ALTs on learners’ SRL, data should be collected and analyzed at all SRL phases (Bellhäuser et al., 2022; Roll & Winne, 2015). In this context, the self-regulation process model (Schmitz, 2001; Schmitz & Wiese, 2006) is a valuable analytical framework since it considers the dynamic nature of SRL, the importance of feedback, and the significance of SRL strategies. This study investigates learner support at various stages of the SRL process over time, employing a digital tool. Due to the learning setting, learners were given a total of one week to complete the tasks. Thus, to measure the dynamics of the SRL process over time, this study was established with a one-week learning cycle as the unit of time. Personalized and timely feedback was provided, both before (pre-actional) and after (post-actional) task completion, to assist learners as necessary.

Given the need of a better understanding of the SRL learning process, the following quantitative research questions were addressed in our study:

- **Research question 1**: How does the weekly development of task-related (meta-)cognitive, motivational, and emotional aspects of learners’ SRL unfold during the *pre-actional phase* over the course of a semester while using an ALT?
- **Hypotheses 1a:**
- Since studybuddy adaptively supports students during the pre-actional phase, we expect an increase in their metacognitive activity (planning), motivational (self-efficacy, task value, goal orientation) as well as positive emotional state (enjoyment), while expecting a decrease in negative emotional state (anger) over time.
- **Hypotheses 1b:**
- Furthermore, we hypothesize that positive motivational and emotional states positively predict planning activity.
- **Research question 2**: How does the weekly development of task-related (meta-)cognitive and emotional aspects of learners’ SRL unfold during the *post-actional phase* over the course of a semester while using an ALT?
- **Hypotheses 2a:**
- As studybuddy adaptively supports students during the post-actional phase as well, we hypothesize an increase in metacognitive activity (organization, monitoring) and emotional state (enjoyment), while expecting a decrease in negative emotional state (anger) over time.
- **Hypotheses 2b:**
- We assume that positive emotional states positively predict monitoring and organization.

A deeper understanding of the SRL process includes the question how learners accept and interact with feedback. While many tools provide automated feedback to learners, there is little evidence on how learners accept and interact with automated feedback (Ifenthaler et al., 2021). The qualitative research questions thus are as follows:

- **Research question 3:** How do students perceive timely, personalized feedback from an ALT, in relation to their SRL processes?
- **Sub question 1:** How do learners perceive and integrate feedback from studybuddy during the pre-actional phase, and how does it inform their subsequent actional practice?
- **Sub question 2:** How do learners perceive and describe feedback from studybuddy during the actional phase?
- **Sub question 3:** How do learners perceive and use feedback from studybuddy during the post-actional phase to inform their next learning cycle?

## Method

**Fig. 3:**
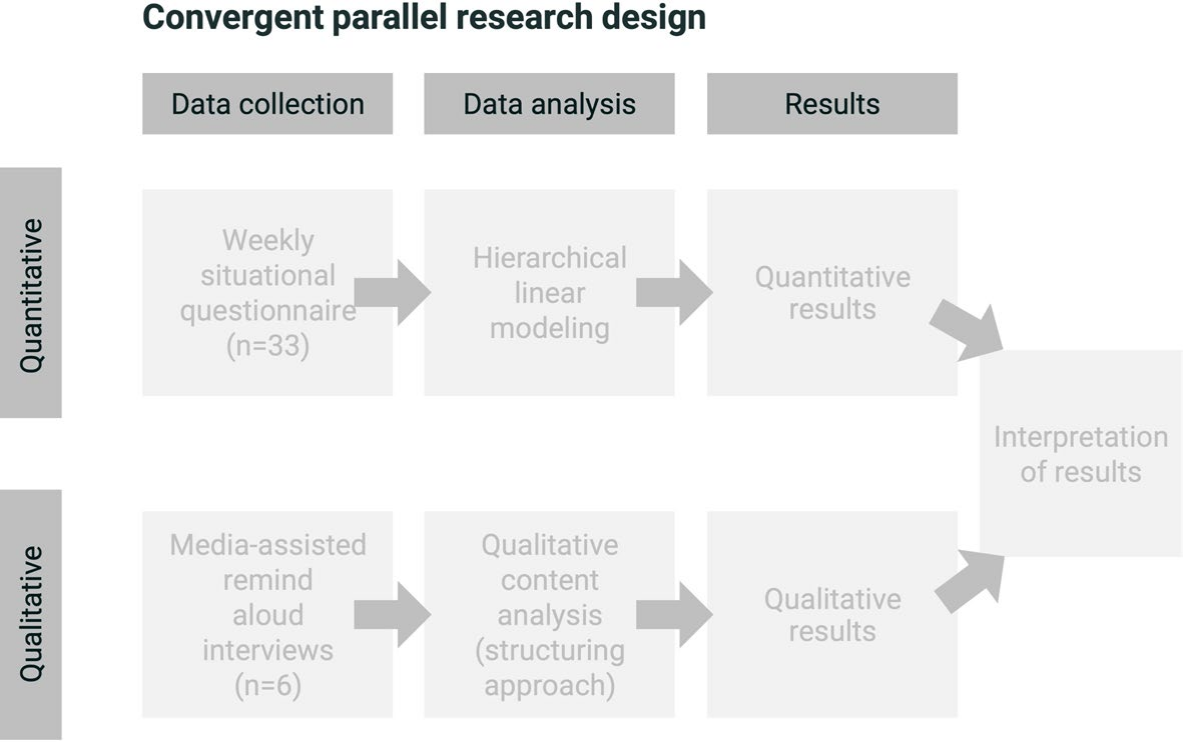
Convergent Parallel Research Design

The present study used a mixed-methods design, specifically a convergent parallel research design (Creswell and Plano-Clark, 2018), to combine qualitative and quantitative strands of research (Fig. 3). This approach allowed us to address the need for more qualitative studies in SRL research (Pekrun, 2020), and provided both broad and deep insights into the development and promotion of SRL through a digital tool (Anthonysamy et al., 2020; Kelle, 2015; McCardle & Hadwin, 2015).

### Participants

A concurrent mixed method sampling was conducted to collect and analyze both qualitative and quantitative data simultaneously (Teddlie & Yu, 2007). The quantitative sample consisted of 33 students with a mean age of 26.3 (*SD* = 3.89) from a university in Switzerland. The students were all undergraduates who were taking a methods course as part of their educational science studies. Of these 33 students, 29 students were female (87.9%) and 4 students were male (12.1%). This gender ratio is typical for educational science programs at Swiss universities. To gain deeper insights relevant to the research interest the case selection for the qualitative sub-study was based on the principle of purposeful or purposive sampling, which involves using a qualitative sampling plan (Schreier, 2017). The selection of students to be interviewed was based on their academic performance and study experience, both factors likely to be related to the phenomenon of interest, as this has been empirically demonstrated in previous research (e.g., Gerholz, 2012). To construct a qualitative sampling design, the factors were combined using the criteria outlined in Appendix B. For each combination of factor expressions, one case was randomly selected, resulting in the selection of six female students from the seminar for the qualitative interviews. The researchers’ university provided ethics approval. The students were informed of the study’s purpose and gave their informed consent. The data collection procedures (qualitative and quantitative) were tested with pilot studies. All data provided by the students were anonymized.

### Procedure

**Fig. 4:**
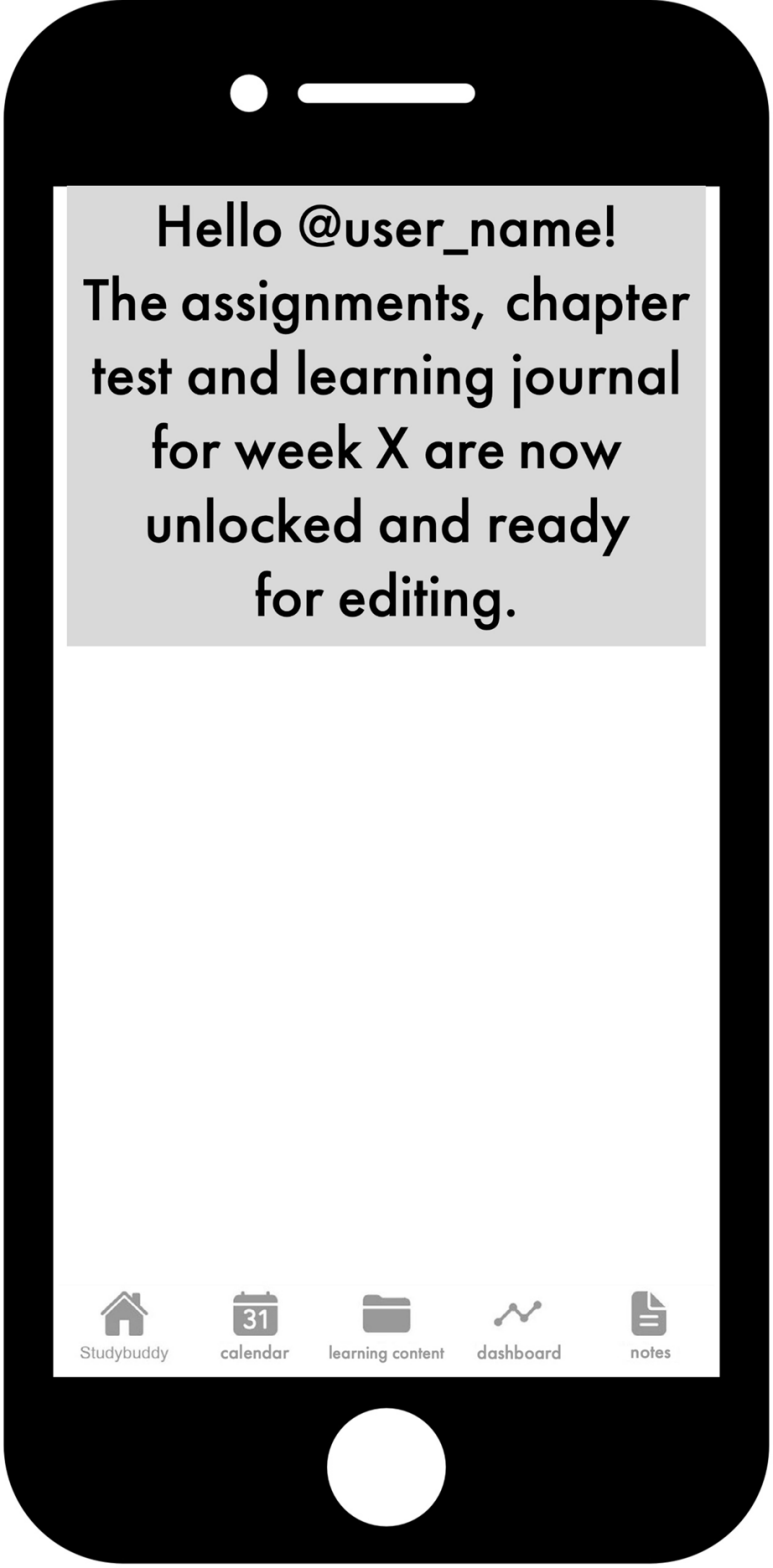
Getting a new task by studybuddy

The context of the study is a mixed-methods course at the authors’ university. The course covers various fundamental positions in the theory of science, as well as the application of different research methods, and comprises a total of 5 ECTS credits. To develop the learning content of the research module in a sustainable way and promote students’ SRL, the course was designed as a Flipped Classroom. In this way, the students were guided to manage their learning process independently, take responsibility for their learning, and that of the entire learning group. Additionally, they were encouraged to develop the seminar content on their initiative, plan their approach independently, and had the opportunity to actively participate in the course’s design. The methods course was structured for students to listen to a podcast weekly over a 12-week period in preparation for the seminar session and read a text. They were also required to complete a chapter test each week. During the face-to-face session, the prepared content was discussed in groups, and the results of the chapter test were reviewed. As part of the seminar, students were asked to install the “studybuddy” app that the authors developed, which provided them with various weekly prompts. The course followed the same format each week:

After each class, students were notified that new tasks awaited them (Fig. 4). The students were then asked to review the tasks for the upcoming week and confirm their understanding by clicking the “Ok, I have an overview” button in the app.

**Fig. 5:**
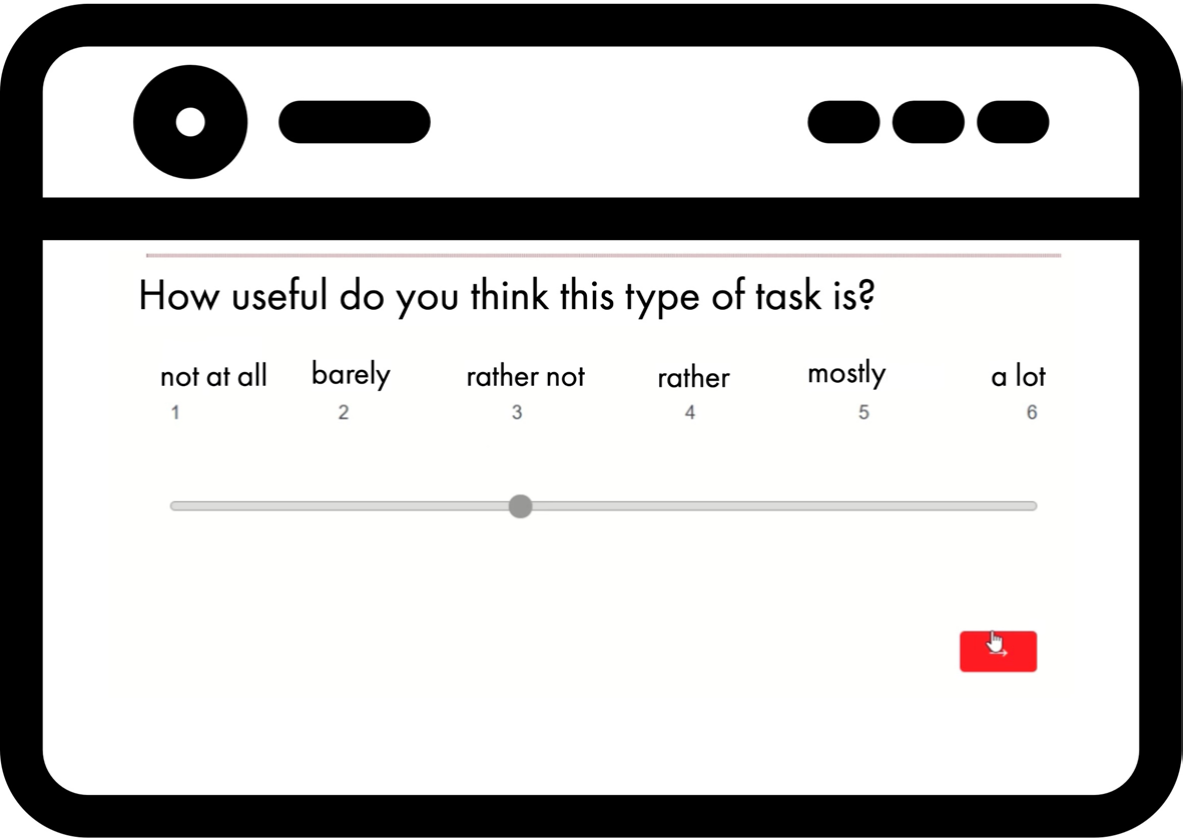
Weekly reflection on SRL

Once the learners had reviewed the tasks (pre-actional phase), the app prompted them to reflect on their motivational and emotional state, as well as their metacognitive activity, in relation to the upcoming task (Fig. 5). This measured the relevant data for the pre-actional phase and provided it to studybuddy.

**Fig. 6:**
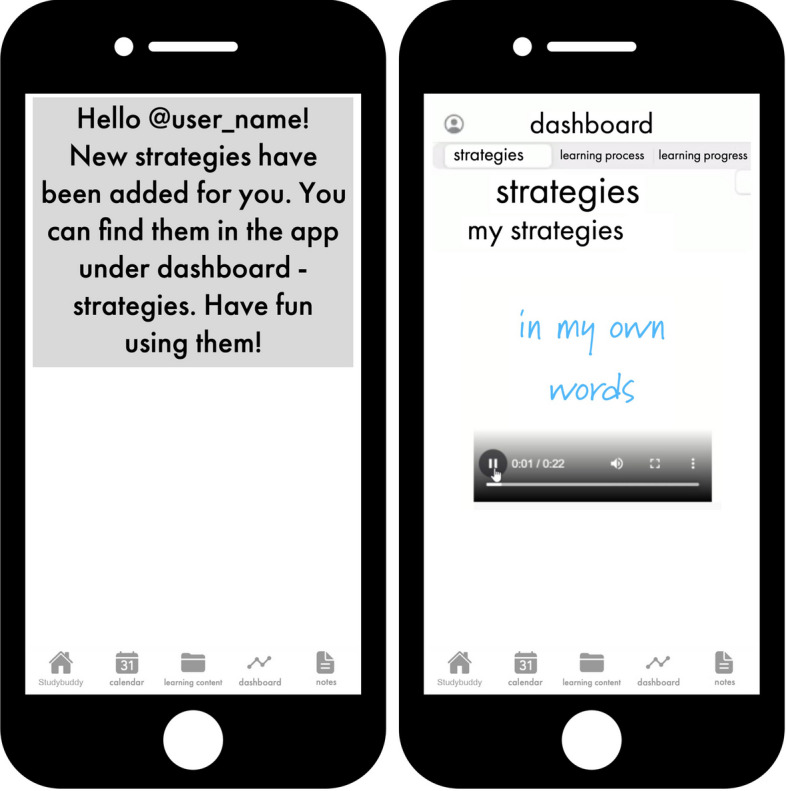
Getting a new strategy suggestion by studybuddy (pre-actional)

If a student did not exceed a specific threshold, the app automatically prompted them with an appropriate regulation strategy. For instance, in the context of the task’s value, learners were asked: “How useful do you think this type of task is?”. If the student ticked one of the four lowest values (“not at all”, “barely”, “rather not”, or “rather”) on the 6-point Likert scale, a regulation strategy was automatically suggested (Fig. 6).

**Fig. 7:**
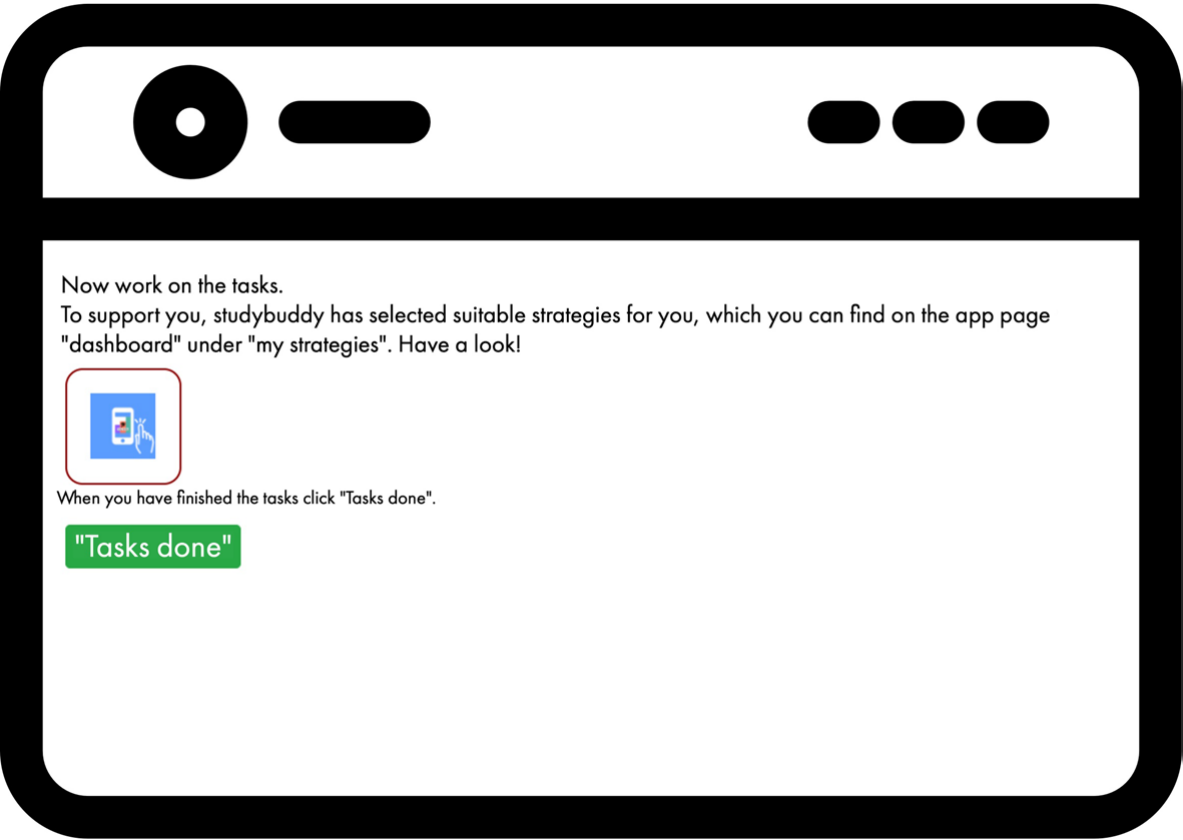
Working on upcoming task

Along with the push message, a notice also appeared in the digital tool (Fig. 7), encouraging learners to begin working on their tasks and unlocking a button that they could press after completing the assignments to indicate their completion.

**Fig. 8:**
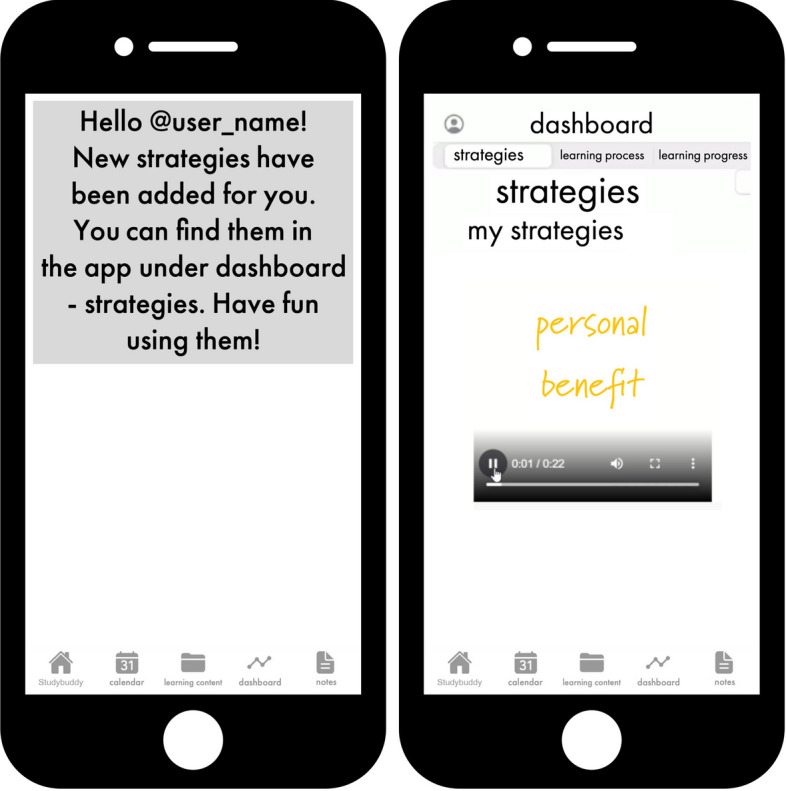
Getting a new strategy suggestion by studybuddy (post-actional)

After completing their weekly tasks, which included literature study, podcasts, a learning journal and chapter tests, students could click on the “Task completed” button in the digital tool. Following the completion of their tasks (post-actional phase), students were then asked to reflect on their cognitive and metacognitive activities, as well as their emotional and motivational state in relation to the completed tasks. This measured the relevant data for the post-actional phase and provided it to studybuddy. Again, if a student did not exceed a specific threshold, the app automatically prompted them with an appropriate regulation strategy (see Fig. 8).

**Fig. 9:**
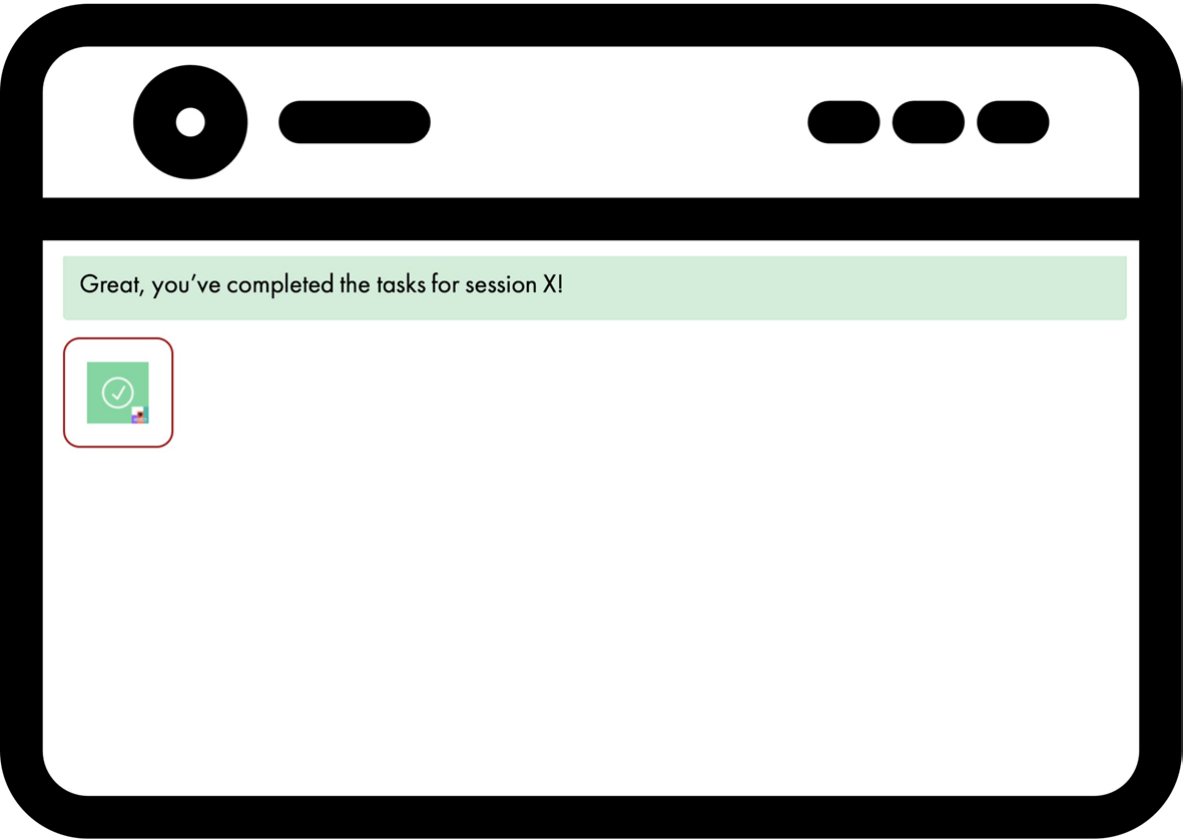
Successful completion of task

In addition to this push message, the digital tool displayed the message that all tasks had now been successfully completed (Fig. 9).

**Fig. 10:**
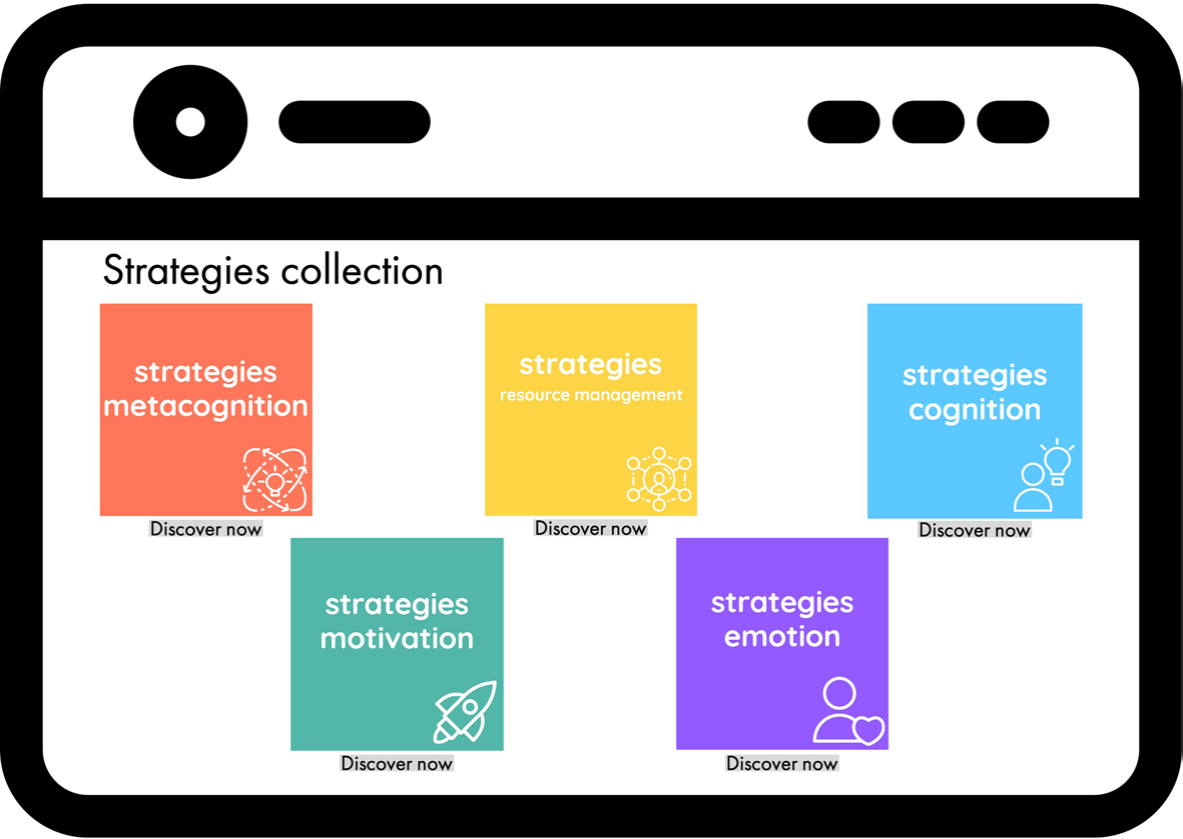
Consulting the strategy collection

Students were also encouraged to consult the strategy collection as needed, such as when personal strategies were not provided that week, when the strategies provided were not helpful, or when unforeseen challenges arose during learning (Fig. 10).

**Fig. 11:**
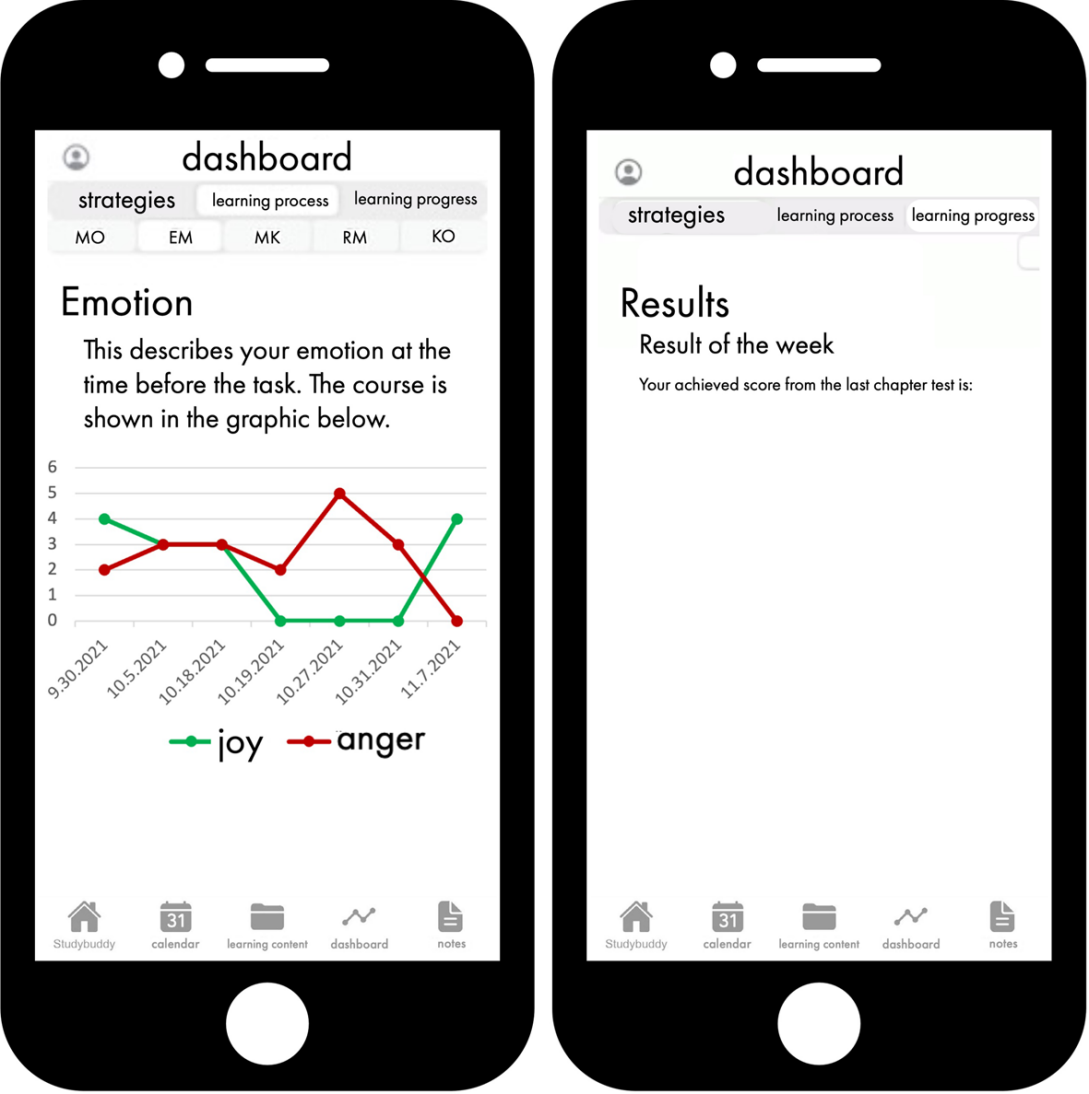
Studybuddy dashboard

At the same time, learners were informed that studybuddy displays information from two questionnaires (completed before and after solving the task) in the app’s dashboard under “learning process”. This allowed learners to easily monitor their progress in areas such as cognitive and metacognitive regulation, emotional and motivational state, and resource management at any time. Additionally, students could view their chapter test results and current overall score (Fig. 11).

### Measures

#### Quantitative measures

Throughout a 9-week period, a set of single items was used in a weekly questionnaire, which has been reported to have adequate psychometric properties and can serve as a suitable alternative to long scales when they are not applicable (Gogol et al., 2014). A time unit consisted of one week, during which students could independently choose when and how long they wanted to spend on the preparation (pre-actional), execution (actional), and follow-up (post-actional) of the tasks. The quantitative measurements were taken at the beginning of the pre-actional phase and the end of the post-actional phase (for details, see Chapter 5.2). Appendix B gives an overview of the measures that were collected before and after studying. All items were adopted from established dispositional questionnaires. For accuracy, we selected the items that best represented each scale (based on the criteria “face validity” and “convergent validity”; Allen et al., 2022; Goetz et al., 2013; Martin et al., 2015, 2020; Schmitz & Wiese, 2006) and had been used in our previous research (Mejeh & Held, 2022). We assessed the reliability of the measurements for each item by computing separate means for odd and even weeks (split-half reliability) and then correlating these means across subjects (Liborius et al., 2019; Schmitz & Wiese, 2006; Theobald & Bellhäuser, 2022). The split-half coefficient for each variable is reported in parentheses in Table 1 and 2. The use of cognitive and metacognitive strategies was measured using one item each, specifically for the subscales of Planning, Monitoring, and Organization in the “Inventory for the Measurement of Learning Strategies in Academic Studies” (LIST; Wild & Schiefele, 1994). To measure motivation, we used the On-Line Motivation Questionnaire (OMQ; Boekaerts, 2002) and included two items for each of the constructs: subjective competence (self-efficacy), personal relevance (task-value), and learning intention (goal-orientation). Finally, emotional state was captured using one item each from the enjoyment and anger scales of the “Achievement Emotion Questionnaire” (AEQ; Pekrun et al., 2005). All items were rated on a 6-point Likert scale ranging from 1 (not at all) to 6 (very much).

**Table 1:** Means, standard deviations, correlations with confidence intervals, and reliabilities of the variables (pre-actional)

Variables	*M*	*SD*	1	2	3	4	5	6
1. Planning	4.43	0.86	(0.95)					
2. Subjective competence	4.19	0.61	.23**	(0.93)				
			[.12, .34]					
3. Personal relevance	4.53	0.74	.53**	.38**	(0.97)			
			[.44, .62]	[.27, .48]				
4. Learning intention	4.24	0.80	.47**	.40**	.65**	(0.96)		
			[.37, .56]	[.29, .50]	[.58, .72]			
5. Enjoyment	3.58	0.94	.31**	.50**	.42**	.38**	(0.95)	
			[.19, .42]	[.40, .58]	[.31, .52]	[.27, .48]		
6. Anger	3.13	0.99	-.18**	-.39**	-.36**	-.21**	-.47**	
			[-.31, -.05]	[-.49, -.27]	[-.47, -.25]	[-.33, -.08]	[-.56, -.36]	(0.65)

*M* and *SD* are used to represent mean and standard deviation, respectively. Values in square brackets indicate the 95% confidence interval for each correlation. * indicates *p* < .05., ** indicates *p* < .01. Split-half reliability is displayed in parentheses on the diagonal

**Table 2:** Means, standard deviations, correlations with confidence intervals, and reliabilities of the variables (post-actional)

Variable	M	SD	1	2	3	4
1. Enjoyment	3.58	0.93	(0.95)			
2. Anger	3.17	1.01	-.38**			
			[-.49, -.27]	(0.79)		
3. Monitoring	4.14	1.04	.26**	.07	(0.94)	
			[.13, .37]	[-.07, .20]		
4. Planning	4.12	1.32	.16*	.09	.55**	
			[.03, .28]	[-.05, .22]	[.45, .63]	(0.93)

*M* and *SD* are used to represent mean and standard deviation, respectively. Values in square brackets indicate the 95% confidence interval for each correlation. * indicates *p* < .05. ** indicates *p* < .01. Split-half reliability is displayed in parentheses on the diagonal

#### Qualitative measures

In consideration of the cyclical nature of feedback a media-assisted remind aloud interview (Knorr, 2013) as a subversion of the stimulated recall method was conducted twice with the selected students (*n* = 6) to collect recollections of thoughts and actions that had already taken place during the handling of feedback provided by studybuddy. Students were prompted to speak out any thoughts they had upon receiving feedback during the week. As the use of media can serve a supportive function in relation to the remembering process (Henderson & Tallman, 2006), students were provided with a comprehensive overview of the weekly feedback course, with each feedback presented in its original output format and accompanied by a brief situating text. Everything was presented in the form of a PowerPoint presentation to the students. The recall process was intentionally kept unstructured to encourage participants to express their thoughts. In this way, they were able to recall and verbalize the thoughts they had while receiving, using, and dealing with the feedback. To promote the recollection of action-justifying statements, direct inquiries were made about participants’ specific thoughts during the feedback activity (see Appendix B) (Knorr, 2013). Students were informed in advance about the scientific purpose of the verbal data, and the data material is available in the form of audio or video files (screen recording).

### Data analysis

#### Quantitative Analysis

We used hierarchical linear modeling to analyze the data, which allowed to differentiate between within-subject and between-subject effects. Additionally, this approach accommodated multiple observations per person, different numbers of observations between participants, and non-equidistant times of measurements, while providing model estimates for missing data. Therefore, a sample size of *N* = 33 students corresponds to having 33 upper-level units in a multilevel design, which is acceptable for the present study, as demonstrated by previous research (Martin et al., 2015), despite being small for single-level designs.

To explore the development and the relation of the variables, a baseline model (model 1) was fitted, which contained only the intercept. Next, a random intercept model was fitted, which included the varying of intercepts across students and time as predictor (model 2). To account for individual differences, random intercepts and random slopes were incorporated in the analysis, allowing for variation in intercepts and slopes across students (model 3). In model 4, we included motivational and emotional time-varying variables as predictors to estimate metacognitive activity during the pre- and post-actional phases. To test our hypotheses, we used one-sided tests and set significance levels at 0.05 for all analyses. All linear mixed-effect models were run using the nlme package (v3.1.162; Pinheiro et al., 2023).

Missing data is a prevalent issue in longitudinal studies, particularly with more frequent measurements. To address missing data, we performed a maximum likelihood estimation (Allison, 2010; Pratama et al., 2016; Schafer & Graham, 2002; Velicer & Colby, 2005). In our study, missing data occurred due to voluntary participation and absences caused by illness during the survey period. The number of missing values on the dependent variables ranged between 1.4% and 18.5% and on average 94% of the students were filling out the weekly questionnaire. All analyses were conducted in R (R Core Team, 2023).

#### Qualitative analysis

A qualitative content analysis using a structuring content analysis approach (Mayring, 2015) was conducted focusing on the perceptions and actions described by the students in response to feedback provided by studybuddy. The audio material was transcribed according to Fuss and Karbach’s (2014) rules. It was transcribed verbatim with minor language editing. To ensure the anonymity of the participating students, a unique code was assigned to each participant. The assigned codes did not reveal the identity of the individual participants but enabled the assignment of a person to a statement.

The data was categorized theory based using Schmitz’s (2001) process model of self-regulated learning, with the main thematic categories: situational conditions, task, emotions, goals, motivation, planned strategy deployment (pre-actional phase); learning strategies / volition, time, monitoring, performance (actional phase) and evaluation / reflection / comparison, emotions, strategy modification, goal modification (post-actional phase). An additional category of “well-being” was inductively added to the categories in the pre-actional phase based on frequent mentions in the data (Mayring, 2015). The full category system can be found in Appendix C. Each sentence unit was coded per occurrence of a condition, and both interpretations, evaluative statements, or understanding of content were included in the analysis. A text segment could be assigned to a code more than once. The software MAXQDA was used for the coding process. To ensure intersubjective comprehensibility, the first and second author conducted and discussed the coding of two interviews together (Kuckartz, 2018). In addition, an inter-rater reliability for the two interviews was calculated, which can be considered as good with a resulting kappa value of 0.82 (Döring & Bortz, 2016).

## Results

In the following section, we present both quantitative and qualitative results in a side-by-side comparison (Creswell & Plano-Clark, 2018). We follow the order of the quantitative questions and provide matching qualitative findings immediately following the quantitative results for each question. The qualitative results collected for the actional phase will be allocated to either the pre-actional or post-actional phases based on the relevance and alignment of the content.

### Descriptive statistics

Tables 1 and 2 display the means, standard deviations, correlations, and reliabilities (in parentheses) for the variables in both the pre- and post-actional phases. As expected, all variables showed significant correlations in the expected direction across measurement points.

In the pre-actional questionnaire, students reported their levels of enjoyment (ICC = 58%), anger (ICC = 36%), subjective competence (ICC = 40%), learning intentions (ICC = 63%), personal relevance (ICC = 63%), and planning strategies (ICC = 55%) for the upcoming task. In the post-actional questionnaire, students reported their levels of enjoyment (ICC = 47%), anger (ICC = 25%), monitoring (ICC = 55%), and organization (ICC = 57%). While the explained variance varies across variables, we can assume relatively equal variance explanation both between and within individuals (i.e., between weeks) in principle.

### Weekly development of SRL

To investigate the development of task-related (meta-)cognitive, motivational, and emotional aspects of learners’ SRL, separate linear mixed models were conducted (H1a, H2a). The model parameters are listed in Table 3 and 4, and the goodness-of-fit for linear changes in all variables studied is listed in Appendix D. The following results refer to the best fitting model (bold marked in Tables D1-D5 in Appendix D^1^).

**Table 3:** Weekly development of the SRL components (pre-actional)

	Planning		Subjective competence		Personal relevance		Learning intention		Enjoyment		Anger	
Predictors	B	β	B	β	B	β	B	β	B	β	B	β
Intercept	4.45 ^***^	-.06	4.08 ^***^	.01	4.62 ^***^	-.07	4.17 ^***^	-.05	3.51 ^***^	-.06	3.07 ^***^	-.04
Time	-.01	-.04	.03 ^*^	.11	-.03 ^*^	-.10	.01	.02	.00	.01	.01	.03
Random Effects												
σ^2^	.31		.21		.18		.19		.42		.74	
τ_00_	.45 _code_		.07 _code_		.37 _code_		.53 _code_		.27 _code_		.12 _code_	
τ_11_	.00 _code.time_		.00 _code.time_		.00 _code.time_		.01 _code.time_		.01 _code.time_		.00 _code.time_	
ρ_01_	-.24 _code_		.50 _code_		-.01 _code_		-.45 _code_		-.01 _code_		.45 _code_	
N	33 _code_		33 _code_		33 _code_		33 _code_		32 _code_		31 _code_	
Marginal R^2^ / Conditional R^2^	.001 / .600		.011 / .446		.009 / .704		.000 / .712		.000 / .530		.001 / .270	

* indicates *p* < .05. ** indicates *p* < .01. *** indicates *p* < .001. 95% confidence intervals of standardized beta coefficient in parentheses. σ^2^ indicates the residual variance. τ_00_ indicates the variance of the intercepts. τ_11_ indicates random slope variance. ρ_01_ indicates random slope intercept correlation

**Table 4:** Weekly development of the SRL components (post-actional)

	Monitoring		Organisation		Enjoyment		Anger	
Predictors	B	β	B	β	B	β	B	β
Intercept	4.03^***^	-.09	4.19 ^***^	-0.15	3.53 ^***^	-0.04	3.07 ^***^	-0.06
Time	.00	.01	-0.06 ^*^	-0.11	0.00	0.01	-0.00	-0.00
Random Effects								
σ^2^	.49		.74		.38		.64	
τ_00_	.65 _code_		1.21 _code_		.52 _code_		.36 _code_	
N	33 _code_		33 _code_		32 _code_		31 _code_	
Marginal R^2^ / Conditional R^2^	.000 / .569		0.010 / 0.626		0.000 / 0.578		.000 / .359	

* indicates *p* < .05. ** indicates *p* < .01. *** indicates *p* < .001. 95% confidence intervals of standardized beta coefficient in parentheses. σ^2^ indicates the residual variance. τ_00_ indicates the variance of the intercepts

### During pre-actional phase

To test the first hypothesis (H1a), separate multilevel models were conducted. The results indicated that during the pre-actional phase, there was a significant increase in the subject competence of the students (b = 0.11, t(241) = 2.26, p = 0.02). Conversely, the personal relevance of the task significantly decreased (b = -0.10, t(237) = -2.62, p = 0.009). Furthermore, the random effects correlation reveals a moderate positive relationship for subjective competence, whereas only a minimal negative relationship is observed for personal relevance. There were no significant effects of time found for the remaining variables, including planning, learning intention, enjoyment, and anger.

The qualitative analysis revealed that positive feedback in the pre-actional phase can help alleviate students’ anxiety and uncertainty about the likelihood of success with upcoming tasks, leading to increased self-efficacy. For example, one student reports an increase in her sense of competence through strategy feedback, realizing that she already has a broad repertoire of learning and work strategies. Feedback in the form of results, such as the dashboard display, provides learners with an indication of how well they can handle assigned tasks. However, different types of feedback can also cause confusion, especially if they conflict with self-formulated goals and deviate from them. In such cases, feedback helps students rethink and adjust their expectations and goals:

And uh yeah, if it then shows something completely different than how I felt or how I personally felt, then I also think about uh what do I have to do there or why are these discrepancies there and what does that mean for the next week or for the next time. (S4_1).

Additionally, the analysis suggests that students with a high degree of self-efficacy and confidence in their abilities tend to pay less attention to external feedback while learners who struggle to adapt their learning without external assistance or have doubts about their own abilities may actively seek support and be more receptive to feedback.

The integration of feedback from the pre-actional phase into students’ actional practice during the actional phase is mirrored in their responsiveness to studybuddy’s feedback. This responsiveness can guide them to take immediate concrete actions, such as completing tasks, submitting evaluations, writing reviews, or scheduling appointments with other group members. Specifically, push notifications from studybuddy seem to play a crucial role in directing students’ attention while working on weekly tasks. It was reported that push notifications can effectively capture learners’ attention and generate interest in specific tasks. For instance, one participant promptly checks her learning journal entries against the model solution after receiving a push notification. Another student highlights the significance of continuous feedback from study buddy in helping her stay on track throughout the semester:

Um well, it actually influenced me in the sense that when it was mentioned that the new tasks and the new learning journal had been released. So, when I received this feedback, I usually downloaded the text right away and got an overview or also inserted the new tasks from the learning journal into our group document. (S4_2).

### During post-actional phase

To test the second hypothesis (H2a), the multilevel models were used to examine the effects of time after task processing in the post-actional phase. The results showed partially significant effects. Specifically, the organization of the students significantly decreased (b = -0.11, t(203) = -2.45, p = 0.02), but only when the slopes were fixed in the models. No significant effects of time were found for the remaining variables, including monitoring, enjoyment, and anger.

The qualitative results in the post-actional phase suggest, that if evaluation-based feedback is poor (e.g., results of chapter tests), it can serve as motivation to examine the feedback more closely in the new week, especially strategy feedback. Furthermore, feedback (especially suggested strategies) seems to trigger memory processes in students, enabling students to recall previously internalized learning behaviors. It is also reported that the use of the proposed strategies has a direct impact on the effective use of strategies in solving tasks. Some learners remember an input from the strategy feedback while solving tasks and use it as inspiration for a potential approach. Others report that they were able to expand their passive strategy repertoire through feedback from studybuddy, and that the strategy suggestions served as a motivational boost to think about their own strategies and reflect on their strategy use:

Um so I watch it and then the first thing I think about each time is um if I already know the strategy so if I already have it somewhere in my repertoire. There were so many, numerous strategies prompted and if it served as inspiration then it certainly achieved the goal. So that I have then again thought about the learning path. (S5_1).

However, interest in the suggested strategies seems to decrease towards the end of the semester. The students cite reasons such as habituation, repetition of the same strategy suggestions, already known strategies, too low cognitive activation from the strategies, or technical problems with the tool. Participants also highlighted positive effects on their well-being: When they receive feedback confirming they have achieved what was planned for the previous week, they feel good and start the new week with a positive mindset. In addition, by displaying learning-related variables in the dashboard, students were able to continuously analyze their own learning. The possibility of testing and evaluating their own learning at regular intervals was perceived as very helpful.

Overall, regarding the development of learners’ SRL, hypothesis H1a—that an increase in subjective competence, personal relevance, learning intention, and enjoyment, as well as a decrease in anger, would occur—can only be partially accepted. While there was a significant increase in subjective competence, there were no expected effects for the other variables, and even a significant decrease in personal relevance of the task. Similarly, hypothesis H2a—that an increase in enjoyment, monitoring, and organization, as well as a decrease in anger, would occur—has to be rejected, as we found no expected effects and even a significant decrease in organization.

### Predicting metacognitive activity based on motivational and emotional state

To test our hypotheses on metacognitive activity during the pre-actional (planning) and post-actional (monitoring) phase, we performed multilevel analyses with persons on level 2 and days within persons on level 1. To test the hypothesis regarding the prediction of planning over time in the pre-actional phase (within-person, level 1), we stepwise added the predictors subjective competence, personal relevance, learning intention, enjoyment, and anger (H2a). Similarly, to predict monitoring and organization in the post-actional phase, we stepwise added the variables enjoyment and anger (H2b). Results are reported in Table 5, 6, and 7.

**Table 5:** Planning based on motivational and emotional state (pre-actional)

	Model 1		Model 2		Model 3		Model 4		Model 5		Model 6		Model 7	
Predictors	B	β	B	β	B	β	B	β	B	β	B	β	B	β
Intercept	4.39^***^	-.05	4.45^***^	-.06	3.73^***^	-.07	2.55^***^	-.03	2.24^***^	-.01	2.66^***^	.02	1.87^**^	.05
Time			-.01	-.04	-.01	-.04	-.00	-.00	-.00	-.02	-.00	-.01	.01	.05
Subjective competence					.17	.12	.02	.02	-.01	-.00	-.11	-.11	-.07	-.08
Personal relevance							.39^***^	.30	.28^**^	.20	.23 ^*^	.13	.29^**^	.23
Learning intention									.22^**^	.24	.15	.17	.12	.14
Enjoyment											.16^*^	.22	.23^**^	.27
Anger													.08	.06
Random Effects														
σ^2^	.33		.31		.29		.29		.28		.26		.26	
τ_00_	.43 _code_		.45 _code_		2.08 _code_		.52 _code_		.56 _code_		.46 _code_		.36 _code_	
					.07 _code.SUBC_		.02 _code.SUBC_		.02 _code.SUBC_		.02 _code.SUBC_		.01 _code.SUBC_	
							.01 _code.REL_		.01 _code.REL_		.02 _code.REL_		.02 _code.REL_	
									.01 _code.INT_		.02 _code.INT_		.02 _code.INT_	
											.01 _code.JOY_		.01 _code.JOY_	
													.01 _code.ANG_	
τ_11_			.00 _code.time_		.00 _code. time_		.00 _code.time_		.00 _code.time_		.00 _code.time_		.00 _code.time_	
ρ_01_			-.24 _code_		-.34		-.29		-.34		-.39		-.48	
					-.88		-.09		-.01		-.23		-.19	
							-.59		-.56		-.57		-.45	
									-.15		.32		.04	
											-.23		.15	
													.18	
N	33 _code_		33 _code_		33 _code_		33 _code_		33 _code_		32 _code_		31 _code_	
Marginal R^2^ / Conditional R^2^	.000 / 0.564		.001 / 0.600		.014 / 0.619		.124 / 0.537		.181 / 0.563		.159 / 0.564		.201 / 0.601	
AIC	542.751		545.332		540.471		529.855		526.819		508.395		465.512	

* indicates *p* < .05. ** indicates *p* < .01. *** indicates *p* < .001. 95% confidence intervals of standardized beta coefficient in parentheses. σ^2^ indicates the residual variance. τ_00_ indicates the variance of the intercepts. τ_11_ indicates random slope variance. ρ_01_ indicates random slope intercept correlation

**Table 6:** Organization based on motivational and emotional state (post-actional)

	Model 1		Model 2		Model 3		Model 4	
Predictors	B	β	B	β	B	β	B	β
Intercept	3.94^***^	-.14	4.19^***^	-.15	3.76^***^	-.11	3.17^***^	-.07
Time			-.06^*^	-.11	-.06^**^	-.12	-.06^*^	-.11
Enjoyment					.15	.11	.20^*^	.15
Anger							.14	.11
Random Effects								
σ^2^	.76		.74		0.73		.78	
τ_00_	1.14 _code_		1.21 _code_		1.02 _code_		.92 _code_	
Marginal R^2^ / Conditional R^2^	.000 / .600		.010 / .626		.024 / .593		.030 / .556	
AIC	693.459		689.563		664.944		613.770	

* indicates *p* < .05. ** indicates *p* < .01. *** indicates *p* < .001. 95% confidence intervals of standardized

beta coefficient in parentheses. σ^2^ indicates the residual variance. τ_00_ indicates the variance of the intercepts

**Table 7:** Monitoring based on motivational and emotional state (post-actional)

	Model 1		Model 2		Model 3		Model4	
Predictors	B	β	B	β	B	β	B	β
Intercept	4.05 ^***^							
	-.09	4.03 ^***^						
	-.09	3.62 ^***^						
	-.03	3.28 ^***^						
	-.03							
Time			.00					
	.01	.00						
	.01	-.00						
	-.00							
Enjoyment					.15 ^*^			
	0.14	0.17 ^*^						
	0.16							
Anger							.09	
	.09							
Random Effects								
σ^2^	.49		.49		.45		.47	
τ_00_	.65 _code_		.65 _code_		.49 _code_		.47 _code_	
Marginal R^2^ / Conditional R^2^	.000 / .570		.000 / .569		.021 / .528		.024 / .508	
AIC	623.985		625.942		575.321		528.858	

* indicates *p* < .05. ** indicates *p* < .01. *** indicates *p* < .001. 95% confidence intervals of standardized beta coefficient in parentheses

σ^2^ indicates the residual variance. τ_00_ indicates the variance of the intercepts

### Positive motivational and emotional states: Catalysts for effective planning activity

During the pre-actional phase, personal relevance of tasks (b = 0.23, t(181) = 3.00, p = 0.003) and task enjoyment (b = 0.27, t(181) = 3.09, p = 0.002) were found to predict higher planning activity. Interestingly, including anger about the task as a predictor made the significant association of learning intention disappear. Furthermore, the random effects correlation reveals a moderate negative relationship for personal relevance, whereas only a minimal positive relationship is observed for enjoyment.

The results of the interviews suggest that feedback plays an important role as a guide for students’ planning work. The various feedback provided by studybuddy can motivate students to set their own goals and commit to future learning and work processes. The push notification sent at the beginning of the week, informing learners about upcoming tasks, can be viewed as a symbolic start to a new learning and working week. This notification can play a crucial role in assisting learners in establishing appropriate goals for their learning endeavors.

So, I actually find the, how should I say, the procedure how the whole thing is carried out with the um first get an overview, I actually find this very good because um on the one hand I have the feeling the first impression is important and on the other hand you can um also plan the time a little. Um how do I want to approach this text now, for example? How do I want uh or when do I want to listen to the podcast? How long do the podcasts last? (S1_2).

Moreover, feedback during the pre-actional phase can trigger concrete actions, such as breaking down tasks into smaller sub-tasks, setting timeframes for completing tasks, or choosing approaches for specific tasks. It even seems likely that the structured form of feedback forces learners to think positively about what they will do in the coming learning and working process. Beyond the concrete initiation of actions, feedback through a digital tool can serve as an incentive for individual goal achievement. In particular, the appearance of the green confirmation box in combination with the feedback that all tasks for the week are completed can have a motivating and calming effect. Receiving feedback is described as a kind of positive pressure that increases motivation to achieve good results.

The strategy suggestions seem particularly helpful in overcoming a lack of motivation. Whether students utilize different types of feedback for their planning appears to be influenced by various factors that highlight the individual significance of feedback. These factors include the frequency of feedback, personal organizational and strategic learning styles, available time, individual emotions, judgments, attitudes, beliefs, and convictions. Moreover, the content of the feedback also plays a crucial role, as repeated feedback often tends to be disregarded:

Yes, at the beginning it was something new and the interest was a bit higher. And over time, I felt like I got used to it. Then you don’t always look at them specifically or you skip them or you see: Ah, it’s this again. And then, it’s done. (S1_1).

### Positive emotional states as basis for successful monitoring and organization

During the post-actional phase, task enjoyment was found to predict higher monitoring (b = 0.16, t(183) = 2.30, p = 0.02) and organization (b = 0.15, t(172) = 2.08, p = 0.004), but only when the slopes were fixed in the models.

In general, it seems likely that different types of feedback trigger reflection processes regarding students’ own learning and work. This enables them to retrospectively contemplate and assess their learning journey, identifying factors contributing to both success and failure. Students report to use the information from different types of feedback (especially dashboards) as a mirror of self-perception and to monitor their progress. Assessments from different types of feedback often coincide, which can help the students to better evaluate their own performance. However, it can also happen that the feedback from studybuddy does not coincide with students’ self-perception. The parameters for evaluating their own learning might be understood differently or feedback might not be sufficiently understandable or comprehensible. The comprehensibility, along with factors such as time, significance, appealing design, and relevance, is mentioned as a decisive factor for whether the feedback can stimulate reflection processes. When there is agreement between the feedback (e.g., chapter test score) and students’ own perception, it seem to trigger satisfaction, whereas poor and/or non-coinciding feedback (e.g., a decreasing learning curve in the dashboard or strategy recommendations that do not match their experiences) can lead to frustration, disappointment, uncertainty, lack of motivation, or surprise. The way feedback is presented can also influence students’ affective reaction to it.

But on the other hand, I was also a bit unsure whether my working and learning behavior was particularly suitable for recommending strategies to me. Um, that’s more of an interpersonal thing. Yes, that triggered more uncertainty in me. (S5_2).

With positive feedback, fewer efforts seem to be made to explore the reasons for it, and the direct application or implementation of the feedback may be less pronounced. In general, the qualitative analyses suggest that feedback through studybuddy either led to an immediate adjustment of learning and working behavior, or the feedback could not be integrated into the learning process after receipt.

I found the impulses very useful, and they were good learning stimuli. It made me reflect on my own learning process. But then I also asked myself if maybe more space could be given to it. Or with the app, I thought it was really cool, but sometimes I didn’t have the strategy on my phone I’d need. Or I didn’t know that it was so specific. I just always went to look at it when I was finished. So, I think there could definitely be some room for improvement. But I think the idea is very good and I appreciate the motivation to link it to learning strategies. (S2_2).

Overall, our findings partially support both hypotheses (H1b, H2b) regarding the prediction of (meta-)cognitive regulation among learners. Task relevance and enjoyment were found to have a significant association with upcoming tasks, while enjoyment as a positive emotional state was found to have a significant association with completed tasks.

## Discussion

Our mixed-method study aimed at contribution to a better understanding of the SRL process in a digital learning setting in higher education. We implemented an ALT called studybuddy in a method course and followed the students over one semester. Aiming at a synthesis and interpretation of our results, we will integrate our quantitative and qualitative findings and discuss whether and how we can draw a meta-inference based on them (Creswell & Plano-Clark, 2018).

### Enhancing SRL through an ALT during the pre-actional phase

With our first quantitative research question, we investigated how SRL develops in the pre-actional phase through the support of an ALT. We also examined how students’ planning can be predicted by their motivational and emotional states. Qualitatively, we studied how learners perceive feedback in the pre-actional phase and integrate it into their learning actions.

#### The role of task-related enjoyment and personalized feedback

Qualitative and quantitative results converged in that task-related enjoyment is an important prerequisite for pre-actional planning. Specifically, students seem motivated to solve upcoming tasks by anticipating the outcome, which is perceived as positive feedback even before task completion (indicated by a “green light” from the digital tool). This feedback shows a calming effect on students, consistent with other findings in feedback on SRL research literature (Grawemeyer et al., 2017; Rowe, 2011). Also, the decreasing trend of task relevance that was found in the quantitative data was confirmed in the qualitative research strand. Interviews highlighted the need for personalized feedback to prevent overwhelming students with different feedback from an ALT, which can affect the relevance of upcoming tasks. The interviews also revealed that some students receive excessive support from the tool even when they require less, emphasizing the importance of tailored feedback through educational technologies (Forsyth et al., 2016; Matcha et al., 2019; Tempelaar, 2020; Tsai et al., 2020). However, the negative trend of task relevance may also be attributed to the learning environment, providing important insights into how tasks should be designed in an SRL-conducive environment. It is possible that the tasks lacked variability or that learners did not understand the significance of the tasks (Broadbent, 2017; Dignath & Veenman, 2021; Jia et al., 2023; Perry et al., 2020).

While some results may converge, others require differentiation. For instance, while our qualitative results suggest that the digital tool effectively supports students with low SRL skills, it also reveals that students with higher SRL skills may overestimate their SRL abilities (Dörrenbacher & Perels, 2016; Peverly et al., 2003). This issue has been discussed in detail in relation to capturing SRL processes and relying on self-reported data (Azevedo & Gašević, 2019; Dijkstra et al., 2023; Winne, 2022; Winne & Perry, 2000). Furthermore, quantitative data shows a decrease in task relevance over time, while qualitative interviews suggests that task relevance is maintained when the digital tool is actively utilized, such as when retrieving suggested strategies. While digital tools like studybuddy can greatly assist learners in focusing their attention on tasks (Chen & Huang, 2014; Verbert et al., 2013), it is important to note that the appropriate acceptance of digital technology in educational settings by users is crucial, as discussed in the Technology Acceptance Model (Marangunić & Granić, 2015). Thus, fostering learners’ digital literacy seems essential in addressing this challenge, which again requires developing metacognitive, resource management, and motivational strategies accordingly (Anthonysamy et al., 2020).

#### Predicting planning activity: Interindividual differences in motivation and emotion

The results from the two strands of methods revealed different insights regarding the role of learning intention during the pre-actional phase on planning. The multilevel models did not demonstrate any significant effects of learning intention on planning and the interviews revealed a more nuanced explanation for this finding. Specifically, the push messages of the digital tool seem to have varying effects on different students, indicating interindividual differences. Again, this highlights the importance of designing and using learning technology in an adaptive and personalized way (Broadbent & Poon, 2015). This finding is further supported by the result that the notifications seemed to act as a trigger for some learners but not for others, emphasizing the importance of taking individual differences in learning into account.

Additionally, interindividual differences, and consequently the role played by the digital tool in students’ learning process, are reflected in the various random-slope intercept correlations. The correlation for personal relevance demonstrates a negative relationship, suggesting that learners who perceive high personal relevance in the tasks are more likely to benefit from the tool. Conversely, for enjoyment and subjective competence, the opposite holds true: learners with higher initial levels in these areas appear to receive more support from the digital tool.^2^ Moreover, the statistical effect of learning intention as a goal orientation diminished when anger was added to the model, emphasizing the significant interplay between emotions and motivations for SRL (Azevedo et al., 2017; Duffy & Azevedo, 2015; Mega et al., 2014; Moos & Azevedo, 2008). Similarly, while no associations were found in the quantitative data, the students reported that positive feedback can reduce anxiety and uncertainty about their likelihood of success on upcoming tasks, and increase their self-efficacy. This report is for example consistent with the findings of Núñez-Peña et al. (2015) who were able to show that perceived usefulness of feedback reduced the negative impact of anxiety on students’ academic achievement.

### Enhancing SRL through an ALT during the post-actional phase

In our second quantitative research question, we investigated how SRL develops in the post-actional phase through the support of ALT. We also examined how students’ monitoring and organization can be predicted by their emotional states. Qualitatively, we studied how learners perceive feedback in the actional as well as post-actional phase.

#### The role of enjoyment in successful monitoring

In the post-actional phase it appeared that students monitored their learning less as time passed, but stimulated recall procedures indicated that students monitored more when they received negative feedback. While this discrepancy could be due to the fact that reactions to negative feedback are more pronounced, and that positive feedback may be more challenging for students to handle, it could also suggest that students need support in learning how to effectively use and respond to feedback, regardless of its nature (Hattie & Timperley, 2007; Theobald & Bellhäuser, 2022). This finding is corroborated by the result that the negative association of time with monitoring dissipates when considering the distinct developmental trajectories of the students, as random slopes could not be modeled in this case. Furthermore, task-related enjoyment is positively associated with monitoring. Students indicated that feedback from the digital tool consistently stimulated reflective processes regarding their own learning and work behaviors. These qualitative findings align with the result that task-related enjoyment predicts increased student monitoring. It becomes apparent that the relationship between task setting and strategy utilization must be well-aligned, underscoring the importance of providing students with the opportunity to actively apply the strategies they have acquired in order to solve tasks. Thus, the relevance of a learning environment conducive to SRL, in which learners are also active agents in their learning process, becomes evident (Corno, 2008; Karlen et al., 2020).

#### The importance of active digital tool use

Although the quantitative analyses did not show any significant development of emotional and motivational variables over time, students reported in interviews that feedback from the digital tool on a completed task fostered positive feelings. The qualitative analyses revealed that students needed to actively use the digital tool dashboard to experience task-specific enjoyment in the post-actional phase. This result is in line with previous findings, which suggest that digital tools can support metacognitive regulation if they are actively used and if the feedback can be directly incorporated by the learners into their current learning process (Long & Aleven, 2013; Molenaar et al., 2021; Verbert et al., 2013). Simultaneously, students report that the feedback must be useful and correspond with their self-perception, which is consistent with current research findings (e.g., Lim et al., 2020). In this context, negative emotions such as anger can arise when the feedback is perceived as bad or does not match the self-perception. Moreover, the form in which the feedback is presented by the digital tool also seems to be important (Schumacher & Ifenthaler, 2018; Sedrakyan et al., 2020).

## Implications and limitations

An important theoretical implication from our study is the suitability of the prefabricated learning strategies presented to learners through video in our digital tool. It is possible that these strategies may not adequately fit the specific learning needs of individual learners. This issue is consistent with the idea presented by Pammer-Schindler and colleagues (Pammer-Schindler et al., 2018) that digital platforms may not be tailored enough to students’ individual needs and abilities, leading to lower usage. In future, it would be of interest to develop adaptive and personalized approaches that incorporate and process learners’ needs even more effectively (Park et al., 2022), by for example combining ALTs with natural language processing (Mejeh & Rehm, in press). Methodologically, multilevel models are a valid approach for capturing individual learning trajectories. In this regard, it could be highly advantageous in the future to more closely examine single intra-individual developments and model them with greater specificity. Accordingly, linking nomothetic and ideographic analyses more intensively (Schmitz, 2000), potentially within the context of learning analytics data (Winne, 2017), could significantly enhance the further development of the SRL field. Practical implications arise from our study in that questions of designing SRL-conducive learning environments should be addressed more strongly in the future. Especially in flipped classroom settings in higher education, student engagement and active learning could be addressed, e.g., through appropriate assignments or group work (Kapur et al., 2022). In addition, it is crucial to prioritize the needs of both learners and instructors when designing ALTs. This can be accomplished through participatory research approaches that actively involve users in the development of educational technology. In our study, managing the triad of research, users, and IT development proved challenging, necessitating constant translation processes between the different levels. To guard against undesirable outcomes or failures, it is advisable to conduct multiple pilot tests and validation loops with the ALTs before the actual study.

Also, some limitations of our study must be taken into account. First, the study design can be criticized for lacking a control group, which precludes making definitive claims about the effect of the digital tool. While some SRL variables in our study showed expected development over time and interviews indicated the effectiveness of the digital tool, we cannot attribute the observed effects exclusively to studybuddy. Furthermore, the small sample size limits the statistical power of the study, although different developments in learners were observed. However, due to limitations in model specification, more complex models with random slopes could only be partially implemented. As a result, we had to interpret the models with fixed slopes. Therefore, our study should be regarded as exploratory, and future research with control groups and larger sample sizes will be necessary to specify more complex models and increase the explanatory power for the effects of the digital tool. Secondly, the validity of self-reports can be questioned, and one possible solution is to supplement them with other data based on learning analytics to obtain multimodal perspectives (Greene et al., 2021; Järvelä & Bannert, 2021; Molenaar et al., 2021). Nevertheless, self-reports can provide important insights for SRL development, especially when combined in qualitative and quantitative analyses (McCardle & Hadwin, 2015; Mejeh et al., 2023). A further limitation concerns the delivery of SRL strategies, which currently rely on a relatively simple metric (i.e., learners failing to reach a certain threshold on a 6-point Likert scale). To enhance the delivery of SRL strategies, it would be beneficial to incorporate additional data based on learning analytics for diagnosis in the future (Azevedo & Gašević, 2019).

## Supplementary Information

Below is the link to the electronic supplementary material.

Supplementary fileA (DOCX 283 KB)

Supplementary fileB (DOCX 23 KB)

Supplementary fileC (DOCX 26 KB)

Supplementary fileD (DOCX 60 KB)

## Data Availability

The data that support the findings of this study are available from the corresponding author, [MM], upon reasonable request.

## Footnotes

1 We also tested for underestimation of within-subject variability by estimating models that explicitly accounted for autocorrelation. However, incorporating autocorrelation did not improve our model estimates, and therefore, we only reported the models without autocorrelation in the manuscript.

2 For a complementary eye test, we have attached the respective plots in Appendix D (Figures D1 & D2).
