# Supplementary material for: Effects of adaptive feedback through a digital tool – a mixed-methods study on the course of self-regulated learning
Source: Educ Inf Technol (Dordr). 2024 Mar 2;29(14):1–43. doi: 10.1007/s10639-024-12510-8 (PMC11511727; doi:10.1007/s10639-024-12510-8)
Supplement: Supplementary file 1 — Supplementary fileA (DOCX 283 KB) [file 10639_2024_12510_MOESM1_ESM.docx]

**Appendix A**

The following are five illustrative examples of strategies within each domain: metacognition, resource management, cognition, motivation, and emotion.

**Domain: Metagcognition**


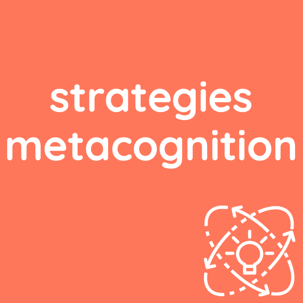


| **Original strategy** | **Adapted strategy** | **Category** | **Video** |
| --- | --- | --- | --- |
| Packe eine unangenehme Aufgabe zwischen zwei angenehmere. (Metzger, 2017) | **Task management**  Consider breaking up an unpleasant task with two more enjoyable tasks today. Aim to create a diversified work plan, and schedule the unpleasant task at a time that suits you best. | Planning Strategies | 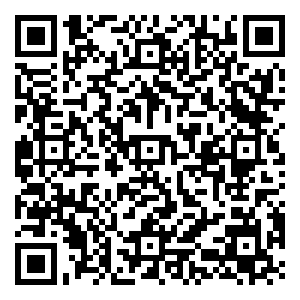 |
| Formuliere selbst Fragen zu dem, was du gerade studierst. (Metzger, 2017) | **Questions upon questions**  Generate your own questions based on the material you've worked on today. Imagine you're creating a test for your classmates on this material - what questions would you ask? | Planning Strategies | 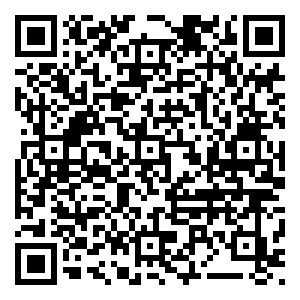 |
| Bringe eine gewisse Regelmässigkeit in die alltäglichen Abläufe hinein, so dass du möglichst rasch auf fas Studium konzentrieren kannst. (Metzger, 2017) | **Consistency**  Today, try to establish a workflow routine that can help you focus more effectively on your tasks or learning materials. | Planning Strategies | 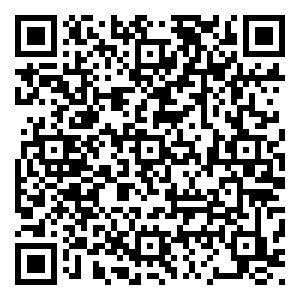 |
| Überlege dir aus Zeitgründen, welche Aufgaben du nur in Gedanken nochmals durchgehst und welche du noch einmal mündlich, schriftlich oder praktisch lösen solltest. (Metzger, 2017) | **Time management**  Consider, in light of time constraints, which material you will mentally review today, and whether you should adopt a different approach such as oral, written, or practical work. | Monitoring Strategies | 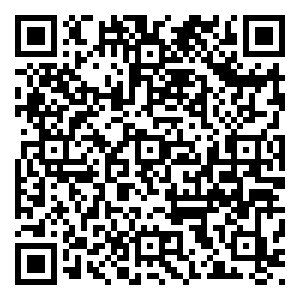 |
| Prüfe ganz gezielt dein Verstehen und Können. (Metzger, 2017) | **Test yourself**  Deliberately test your comprehension and abilities today by using targeted exercises or short quizzes to showcase and verify your understanding as you answer questions. | Monitoring Strategies | 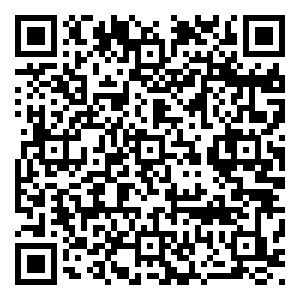 |
| … | **…** | … | … |

**Domain: Resource Management**


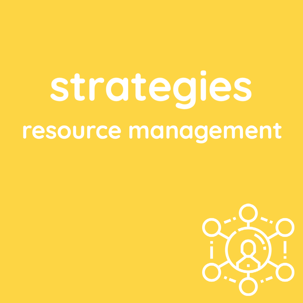


| **Original strategy** | **Adapted strategy** | **Category** | **Video** |
| --- | --- | --- | --- |
| I usually study in a place where I can concentrate on my course work. (Printrich et al., 1991) | **Flow space**  Take a moment today and think: Where is your work space at which can you focus the best? Are you already at your ideal work space or is there a possibility to move there? | Learning environment | 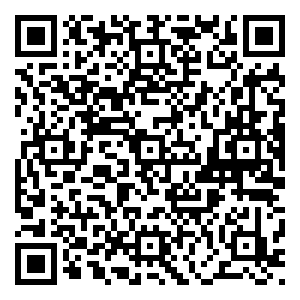 |
| Bemühe dich um Kontakte zu einigen Mitstudierenden im gleichen Semester, so dass ihr euch gegenseitig informieren und unterstützen könnt. (Metzger, 2017) | **Connections**  Make an effort to connect with classmates who are in the same class as you. This way, you can inform and support each other. | Help seeking | 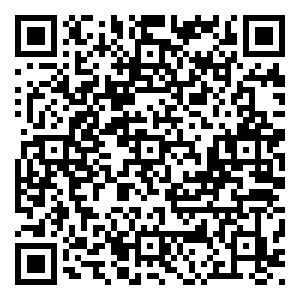 |
| Ziehe eine zusätzliche Quelle bei, also. Z.B. ein Wörterbuch, ein Lexikon, ein anderer Text, ein/e Mitstudierende/r. (Metzger, 2017) | **Peer support**  Communicate with your classmates and ask for help if you are stuck on a task, or just in general regarding any subject matter. | Help seeking | 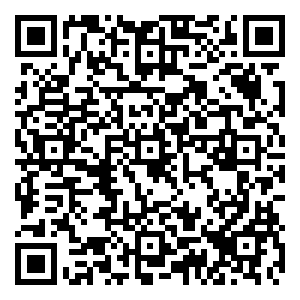 |
| Belohne dich selbst und geniesse den Erfolg. (Metzger, 2017) | **Small successes**  Reward yourself if you have achieved today's learning goals. Even small successes should be celebrated. | Effort | 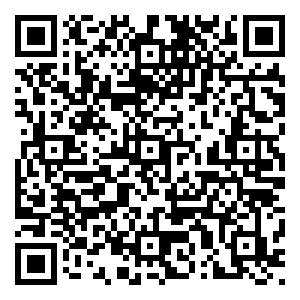 |
| Entspannungsübungen: Schliesse die Augen, atme ganz ruhig und fest durch, zähle innerlich bis zehn, und öffne die Augen wieder. (Metzger, 2017) | **Stop and pause**  Remember to take short breaks throughout your work or study session. Close your eyes, take deep and steady breaths, count to ten internally, and then open your eyes again. | Effort / Attention | 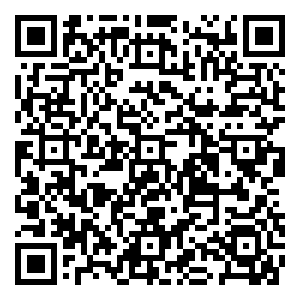 |
| … | **…** | … | … |

**Domain: Cognition**


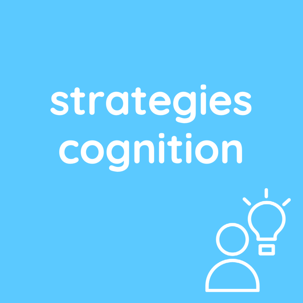


| **Original strategy** | **Adapted strategy** | **Category** | **Video** |
| --- | --- | --- | --- |
| Lese oder löse die Aufgabe nochmals, aber langsamer. (Metzger, 2017) | **Slow down**  Revisit the study material from today by going over it once more. This could involve re-reading the text or attempting the task again. Make sure to take enough time and take breaks intermittently. | Organizational Strategies / Repetition Strategies | 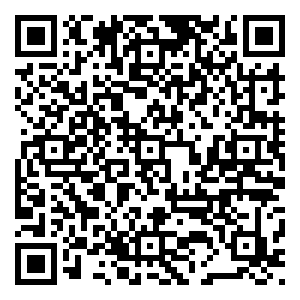 |
| Erzähle das Gelesene oder Gehörte in eigenen Worten. (Metzger, 2017) | **In my own words**  Summarize what you've read and heard in your own words. Consider how you would explain the study material to your classmates. | Organizational Strategies | 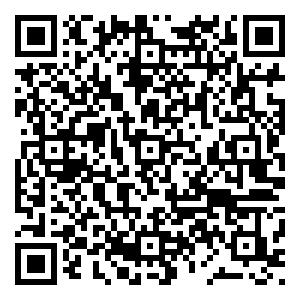 |
| Fasse etwas auf das Wesentliche zusammen. Skizziere das Wesentliche nochmals in Form von Mindmaps oder Concept Maps. (Metzger, 2017) | **Focus on the essential**  Summarize the study material by identifying its core components. Create mind maps or concept maps to outline the key concepts. Research suitable tools that you can use to make mind maps or concept maps, and experiment with different options to find the one that works best for you. | Organizational Strategies | 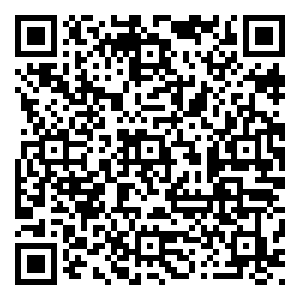 |
| Suche Beziehungen zwischen Neuen und Bekannten, indem due abklärst, was du schon weisst und was du vom neuen Lerninhalt erwartest. (Metzger, 2017) | **Connecting new and old**  Improve your understanding of new material by exploring the relationship between what you already know and what you expect to learn from it. Take the time to clarify your existing knowledge and your expectations for the new content. | Elaboration Strategies | 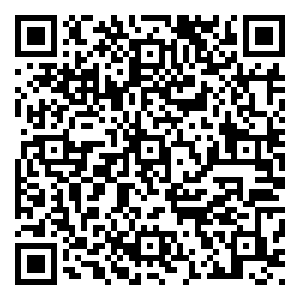 |
| Lese dich in die Thematik schon vor der Lehrveranstaltung ein, damit du in der Lehrveranstaltung erleben kannst, dass du bereits einiges weisst. (Metzger, 2017) | **I know that**  To actively engage during class and build on your existing knowledge, it's advisable to read up on the topic before the lesson if possible. | Elaboration Strategies | 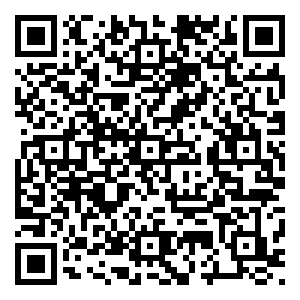 |
| … | **…** | … | … |

**Domain: Motivation**


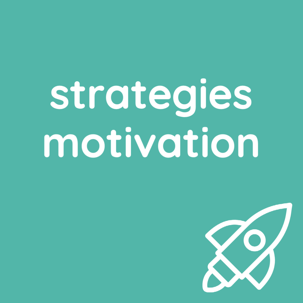


| **Original strategy** | **Adapted strategy** | **Category** | **Video** |
| --- | --- | --- | --- |
| Führe den Erfolg beim Lernen auf deine eigene Anstrengung, nicht auf den Zufall. (Metzger, 2017) | **Success is not luck**  Today, take credit for your success in learning and attribute it to your own efforts rather than to luck. | Self-efficacy | 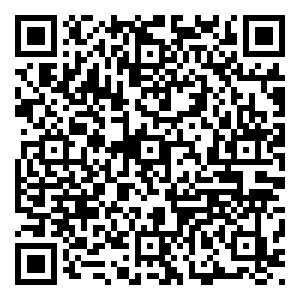 |
| Besinne dich auf deine Stärken und Interessen, denke an persönliche Erfolge zurück. (Metzger, 2017) | **Strengths and accomplishments**  Today, take time to reflect on your strengths, interests, and personal accomplishments. Can you recognize what you have achieved so far? | Self-efficacy | 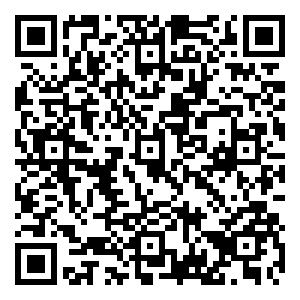 |
| Betrachte möglichst vieles nicht als blosse Pflicht oder gar Bedrohung, sondern als eine Herausforderung. (Metzger, 2017) | **Challenge accepted**  Instead of viewing tasks as burdensome obligations or threats, see them as opportunities for growth and challenge today. | Self-efficacy / Self-motivation | 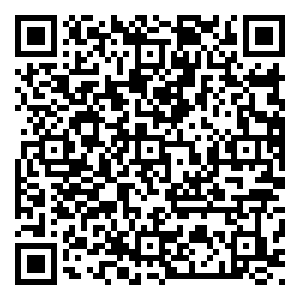 |
| Frage dich selber, ob du einem Lerngegenstand gegenüber positiv eingestellt bist. (Metzger, 2017) | **Attitude**  Today, take a moment to ask yourself if you have a positive attitude towards school. If not, try to identify the reasons why you feel that way. | Value of school | 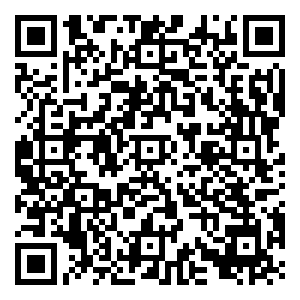 |
| Vermeide unnötigen persönlichen Wettbewerb mt Studienkolleg:innen oder auch im Privatleben. (Metzger, 2017) | **No need for competition**  Avoid personal competition with your classmates or in your personal life. Such situations can often be exhausting and unproductive. | Mastery orientation | 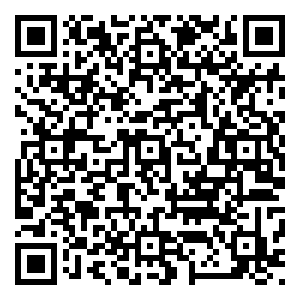 |
| … | **…** | … | … |

**Domain: Emotions**


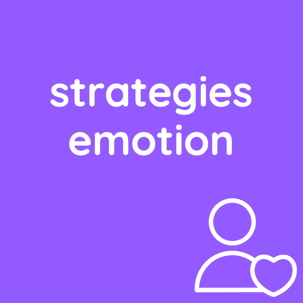


| **Original strategy** | **Adapted strategy** | **Category** | **Video** |
| --- | --- | --- | --- |
| Stelle negative Gedanken positive gegenüber, indem du die Selbstgespräche in eine positive Richtung lenkst. (Metzger, 2017) | **Positive Mindset**  Counter negative thoughts today by consciously directing your self-talk and thought patterns towards positivity. | Joy/Hope/ Anger/Fear | 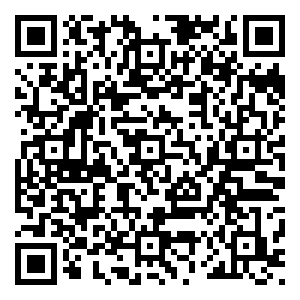 |
| Nutzen Sie die Zeit, um über eine für Sie persönlich emotional belastende Situation zu schreiben, in welcher Sie Ihre eigenen Gefühle unterdrückt, nicht ausreichend gezeigt oder verarbeitet haben (Barnow et al. 2016, S.61) | **Expressive writing**  If you are feeling emotionally overwhelmed today, take a pen and some paper and write down all of your feelings. Don't worry about correct spelling or grammar; your writing flow should not be disturbed. How do you feel after expressive writing? | Joy/Hope/ Anger/Fear | 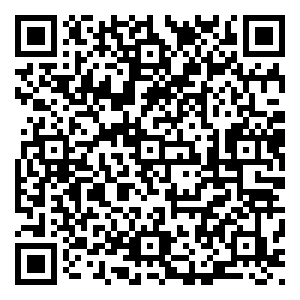 |
| Nehmen Sie die Position eines neutralen Beobachters ein und fragen Sie sich, wie dieser die Situation betrachten würde. (Barnow et al., 2016, S. 63) | **Reappraisal**  After experiencing a frustrating or emotionally charged situation today, take a moment to reflect on it. Try to put yourself in the position of a neutral observer and ask yourself how this person would view the situation. Has your perspective changed? | Joy/Hope/ Anger/Fear | 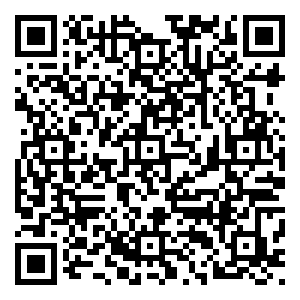 |
| Tagträumen: Versuche zu spüren, was du schon einmal Positives erlebt und gefühlt hast. (Metzger, 2017) | **Memory box II**  Allow yourself a short break after a long day and attempt to feel the positivity that you have previously experienced the last few days. | Joy/Pride/Anger/Fear | 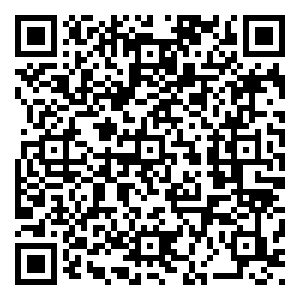 |
| Immunisieren: Stelle dir eine Situation vor, die dich besonders beängstigt, entspanne dich nun und mache dir ein möglichst positives Bild von dieser Situation. (Metzger, 2017) | **Immune II**  Did you experience a particularly frightening or stressful situation today? Take a moment to reflect on that situation and try to relax by visualizing the most positive outcome possible. Repeat this exercise several times until the situation no longer seems frightening or stressful. | Joy/Pride/Anger/Fear | 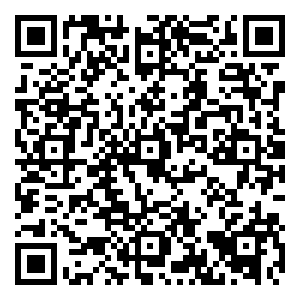 |
| … | **…** | … | … |
