# Supplementary material for: Effects of adaptive feedback through a digital tool – a mixed-methods study on the course of self-regulated learning
Source: Educ Inf Technol (Dordr). 2024 Mar 2;29(14):1–43. doi: 10.1007/s10639-024-12510-8 (PMC11511727; doi:10.1007/s10639-024-12510-8)
Supplement: Supplementary file 2 — Supplementary fileB (DOCX 23 KB) [file 10639_2024_12510_MOESM2_ESM.docx]

**Appendix B - Measurement Instruments**

**B1. Quantitative - Items**

**Inventory for the Measurement of Learning Strategies in Academic Studies (Wild & Schiefele, 1994)**

Planning

1. I think about how to work on the task at hand. *(pre-actional)*

Organization

1. I created brief summaries with the main ideas of the learning material. *(post-actional)*

Monitoring

1. I mentally reviewed the learned material again to see if I retained all the essential information. *(post-actional)*

**On-Line Motivation Questionnaire (Boekaerts, 2002)**

Subjective competence

1. How well do you think you can do this type of task? *(pre-actional)*
2. How well do you expect to do on this task? *(pre-actional)*

Personal relevance

1. How useful do you consider this kind of task? *(pre-actional)*
2. How important do you find it to do well on this sort of task? *(pre-actional)*

Learning intention

1. My goal on this task is to do as well as possible. *(pre-actional)*
2. How much effort are you going to put into this task? *(pre-actional)*

**Achievement Emotion Questionnaire (Pekrun et al., 2005)**

Enjoyment

1. I am looking forward to the task ahead. *(pre-actional)*
2. I was happy about completing the task. *(post-actional)*

Anger

1. The upcoming task annoys me. *(pre-actional)*
2. I was annoyed about completing the task. *(post-actional*

**B1. Quantitative – Split-Half-Reliability**

| ***Scale*** | ***Items*** |  |
| --- | --- | --- |
| Planning *(pre-actional)* | I think about how to work on the task at hand. | *r_sh_* = .91 |
| Organization *(post-actional)* | I created brief summaries with the main ideas of the learning material. | *r_sh_* = .91 |
| Monitoring *(post-actional)* | I mentally reviewed the learned material again to see if I retained all the essential information. | *r_sh_* = .91 |
| Subjective competence *(pre-actional)* | How well do you think you can do this type of task?  How well do you expect to do on this task? | *r_sh_* = .91 |
| Personal relevance *(pre-actional)* | How useful do you consider this kind of task?  How important do you find it to do well on this sort of task? | *r_sh_* = .91 |
| Learning intention *(pre-actional)* | My goal on this task is to do as well as possible.  How much effort are you going to put into this task? | *r_sh_* = .91 |
| Enjoyment *(pre-actional)* | I am looking forward to the task ahead. | *r_sh_* = .91 |
| Enjoyment *(post-actional)* | I was happy about completing the task. | *r_sh_* = .91 |
| Anger *(pre-actional)* | The upcoming task annoys me. | *r_sh_* = .91 |
| Anger *(post-actional)* | I was annoyed about completing the task. | *r_sh_* = .91 |

**B2. Qualitative**

**Factor combination to create the qualitative sample design**

|  | Enrollment  2007-2015 | Enrollment  2016-2017 | Enrollment  2018-2020 |
| --- | --- | --- | --- |
| below grade point average | 7 | 5 | 5 |
| above grade point average | 5 | 4 | 7 |

**Direct inquiries to promote the recollection of action-justifying statements**

I will show you your entire feedback history for week 6-7 below.

*Task:* Look at the feedback and deal with it. While doing this, imagine that you are alone and express aloud any thoughts that are going through your mind at that moment.

*Prompts:*

- What value does this feedback have for you / for your work?
- How do you evaluate your own work / your group work when you see the feedback?
- How do you feel when you see this feedback?
- How do you react to this feedback? (conclusions and actions)
