# Supplementary material for: Effects of adaptive feedback through a digital tool – a mixed-methods study on the course of self-regulated learning
Source: Educ Inf Technol (Dordr). 2024 Mar 2;29(14):1–43. doi: 10.1007/s10639-024-12510-8 (PMC11511727; doi:10.1007/s10639-024-12510-8)
Supplement: Supplementary file 3 — Supplementary fileC (DOCX 26 KB) [file 10639_2024_12510_MOESM3_ESM.docx]

**Appendix C**

| **Thematic categories** | **Description** | **Example** | **Encoding rules** |
| --- | --- | --- | --- |
| **Pre-actional phase** | | | |
| **Situational conditions** | Any parts of the text that refer to the situational conditions which the student is facing. | *"Yes there that is again so. Um rather so a temporal affair also. Whether I really after the Augaben ähm the motivation or strength at all still have to make something for it. And therefore they are really looked at or not depending on the day." (S1_1)* | - Content understanding, as well as interpretations and evaluative statements around the described category  - Sentence units |
| **Task** | Any parts of the text that indicate the selection and handling of upcoming tasks. | *"Um, yes, for me it triggered more that I thought less about the strategies, but rather that I started to think about when I would like to do what. Um because I divide the tasks a bit and um yes that was with me it triggered more so the future thinking always the whole process simply. Ah yes how do, so how do I plan that now." (S3_2)* | (cf. above) |
| **Emotions** | Any parts of the text that indicate the emotional state of the student(s) in relation to the tasks ahead. | *"Um so the feedback last week um. I was very proud of us because it was really good feedback. And um it also encouraged us a little bit to continue and to become even better. And I have the feeling that especially positive feedback in a methodology seminar is a nice feeling. And um. Yes. There you also really want to continue at this level, so to speak." (S1_1)* | (cf. above) |
| **Goals** | All text parts that refer to setting personal goals and the relevance of achieving them. | *"I have to say that I didn't summarize the text last week. And then I said to myself, okay, then you just have to do it again this week, summarize the text. Yes, I didn't have so much time last week and so I just said to myself: So now you invest more, uh summarize the text, then it will be good because it's like the last learning journal now this week and the last tasks that we have to solve, as much as I understood and I just wanted to use my resource management completely so that I can just get the points again." (S6_1)* | (cf. above) |
| **Motivation** | All parts of the text that indicate the motivational state of the students in relation to the upcoming tasks. | *"Since it always went well, the feedback simply confirmed to me that my behavior continued to go well or that it actually worked well for the result and that's why I knew I could continue in this way. So it confirmed like a little bit that I either that it goes well and otherwise I would have also noticed that it does not go well and must change something." (S4_2)* | (cf. above) |
| **Planned strategy deployment** | Any portions of text that indicate the student's planned use of strategy and how they plan to proceed in relation to upcoming assignments. | *"So I actually find the, how should I say, the procedure how the whole thing is carried out with the um first get an overview, I actually find really very good because um on the one hand I have the feeling the first impression is important and on the other hand you can um also plan the time a little. Um how do I want to approach this text now for example? How do I want uh or when do I want to listen to the podcast? How long do the podcasts last?" (S1_2)* | (cf. above) |
| **Wellbeing** | All text parts that refer to personal well-being in relation to solving tasks. | *"It's more motivating when I compare it with other events. Because it was just 1:1. We were in a group, we were supervised. We had feedback. I always knew: Ah, now I'm done. And now I can go further. It's a closer supervision that is also more motivating. So this presence, or. It doesn't matter whether it's virtual or through the tutor, who comes and gives us tips and tells us what's good, what we need to optimize. It's just like a different presence than when I think of other events where it's handled completely differently." (S2_2)* | (cf. above) |
| **Actional phase** | | | |
| **Learning strategies / volition** | All text portions that indicate the use of learning strategies while solving the tasks and the student's approach to solving the tasks. | *"But at the very beginning, for example, I was given the strategy to look at it as a challenge and not as an overload or I don't remember which wording it was. And then I was a bit annoyed because I thought to myself yes, yes, it's okay, I know that. Um and then I pushed it away again so mentally but actually when I was then at the text and like so thought Oh it takes no end there it came back into my mind. I thought: Yes, actually, this strategy has actually it already has its justification. And then it came back to me and um it's like so I take note of it just it happens a valuation and then sometimes it comes back to me through the new work process." (S5_1)* | (cf. above) |
| **Time** | All text parts that refer to the time required and time management in relation to solving the tasks. | *"Then I knew like for me, Ah yeah I want to go back through all the podcast and do a summary for me. That for example. That I then didn't do that immediately, but then had in the back of my mind: Ah I want to do that and then when I had enough time, had a time window, I then implemented that. So also a lot." (S2_1)* | (cf. above) |
| **Monitoring** | All text parts that refer to monitoring processes while solving the tasks. | *"So I always looked at those then, as I said, and then thought about it. So. And then I thought about what makes sense in my everyday life for the application? What is now still which existing resources I can now implement? And I found it very stimulating. So. Because then you just thought about your own learning again." (S2_2)* | (cf. above) |
| **Performance** | All parts of the text that indicate the extent to which students succeed in solving the tasks. | *"So it actually influenced me when I was told that the new tasks and the new learning journal was activated. So when I got this feedback I usually downloaded the text right away and got an overview or also added the new tasks from the learning journal to our group Google Docs." (S4_2)* | (cf. above) |
| **Post-actional phase** | | | |
| **Evaluation / reflection / comparison** | All text portions that indicate the student's assessment of his/her performance based on his/her evaluation criteria and modulated by his/her performance level goal as well as in comparison to the performance of other students. | *"S: I found it valuable, yes. It is also for me a mirror or a confirmation of my self-perception. Because it would have been strange if I had now found: Ah I was mega motivated, so very motivated and it would then have been all down. It would also have been interesting for me as feedback. It is like then also interesting and one can make oneself with this representation or one can, one has it verbildlicht and can one then also better in such a way thoughts to that. Better to that then also reflect and find solutions. Yes." (S2_2)* | (cf. above) |
| **Emotions** | All parts of the text that refer to the students' emotional perception after solving the tasks. | *"You have invested a lot of time there. Um disappointing I think is the wrong word but it's certainly also a bit upsetting so the feedback always too. Or if you have invested a lot of time. And at the same time I find it also always very instructive learning. Or I then get a get feedback on what I have done. And then learn a lot in the process. And I find that very positive. Because it's not... There are few events where you get the same 1:1 feedback on your work. Like this. And that on a weekly basis. So I think it makes a lot of sense and we also learn a lot." (S2_1)* | (cf. above) |
| **Strategy modification** | All parts of the text that refer to adjustments of one's own strategies or concrete actions after solving the tasks and through the initiated reflection. | *"So the implementation is not really there most of the time. I do think about why exactly this message has come or why exactly this strategy is suggested. But then it usually stops. So the real conversion happens with me like not. I have the feeling. Maybe subconsciously but otherwise." (S1_1)* | (cf. above) |
| **Goal modification** | All parts of the text that indicate adjustments of one's goals or concrete actions after solving the tasks and through the initiated reflection. | *"And um yes if it then indicates something completely different than I felt or than I personally felt then I also think about um what do I have to do there or what why are these discrepancies there and what does that mean for the next week or for the next time then." (S4_1)*  *"Um I worry about it. Yes. Do I think about it like the input. But in the positive. So like what can I take up now for me? Yes with the available resources. What do I want to implement now and so. Mhm." (S2_1)* | (cf. above) |
