# Supplementary material for: Effects of adaptive feedback through a digital tool – a mixed-methods study on the course of self-regulated learning
Source: Educ Inf Technol (Dordr). 2024 Mar 2;29(14):1–43. doi: 10.1007/s10639-024-12510-8 (PMC11511727; doi:10.1007/s10639-024-12510-8)
Supplement: Supplementary file 4 — Supplementary fileD (DOCX 60 KB) [file 10639_2024_12510_MOESM4_ESM.docx]

**Appendix D**

Table D1

Model parameters and goodness of fit for linear changes in planning and subjective competence

| *Planning* | Model 1 | | Model 2 | | Model 3 | |
| --- | --- | --- | --- | --- | --- | --- |
| Predictors | Estimates | std. Beta | Estimates | std. Beta | Estimates | std. Beta |
| (Intercept) | 4.39 ^***^ (-0.05) | -0.05 (-0.33 – 0.22) | 4.44 ^***^ (-0.05) | -0.05 (-0.33 – 0.22) | 4.45 ^***^ (-0.06) | -0.06 (-0.34 – 0.22) |
| Time |  |  | -0.01  (-0.03) | -0.03 (-0.12 – 0.05) | -0.01  (-0.04) | -0.04 (-0.14 – 0.06) |
| Random Effects | | | | | | |
| σ^2^ | 0.33 | | 0.33 | | 0.31 | |
| τ_00_ | 0.43 _code_ | | 0.43 _code_ | | 0.45 _code_ | |
| τ_11_ |  | |  | | 0.00 _code.time_ | |
| ρ_01_ |  | |  | | -0.24 _code_ | |
| N | 33 _code_ | | 33 _code_ | | 33 _code_ | |
| Observations | 264 | | 264 | | 264 | |
| Marginal R^2^ / Conditional R^2^ | 0.000 / 0.564 | | 0.001 / 0.568 | | 0.001 / 0.600 | |
| AIC | 542.751 | | 544.162 | | 545.332 | |
| log-Likelihood | -268.376 | | -268.081 | | -266.666 | |
| *Subjective competence* | Model 1 | | Model 2 | | **Model 3** | |
| Predictors | Estimates | std. Beta | Estimates | std. Beta | Estimates | std. Beta |
| (Intercept) | 4.20 ^***^ (0.01) | 0.01 (-0.22 – 0.25) | 4.08 ^***^ (0.01) | 0.01 (-0.22 – 0.25) | 4.08 ^***^ (0.01) | 0.01 (-0.23 – 0.25) |
| Time |  |  | 0.03 ^*^ (0.11) | 0.11 (0.01 – 0.20) | 0.03 ^*^ (0.11) | 0.11 (0.00 – 0.21) |
| Random Effects | | | | | | |
| σ^2^ | 0.22 | | 0.22 | | 0.21 | |
| τ_00_ | 0.15 _code_ | | 0.15 _code_ | | 0.07 _code_ | |
| τ_11_ |  | |  | | 0.00 _code.time_ | |
| ρ_01_ |  | |  | | 0.50 _code_ | |
| N | 33 _code_ | | 33 _code_ | | 33 _code_ | |
| Observations | 275 | | 275 | | 275 | |
| Marginal R^2^ / Conditional R^2^ | 0.000 / 0.401 | | 0.011 / 0.414 | | 0.011 / 0.446 | |
| AIC | 432.236 | | 429.158 | | 427.607 | |
| log-Likelihood | -213.118 | | -210.579 | | -207.804 | |

*Note*. Standardized beta coefficient in parentheses. Model 1 = baseline model, Model 2 = random intercept

Model with time, Model 3 = random intercept random slope model; AIC = Akaike information criterion;

logLik = log-likelihood

Table D2

Model parameters and goodness of fit for linear changes in personal relevance and learning intention

| *Personal relevance* | Model 1 | | Model 2 | | | **Model 3** | | |  |
| --- | --- | --- | --- | --- | --- | --- | --- | --- | --- |
| Predictors | Estimates | std. Beta | Estimates | std. Beta | | Estimates | | std. Beta |  |
| (Intercept) | 4.48 ^***^ (-0.06) | -0.06 (-0.37 – 0.24) | 4.62 ^***^ (-0.06) | -0.06 (-0.37 – 0.24) | | 4.62 ^***^ (-0.07) | | -0.07 (-0.38 – 0.24) |  |
| Time |  |  | -0.03 ^**^ (-0.10) | -0.10 (-0.17 – -0.02) | | -0.03 ^*^ (-0.10) | | -0.10 (-0.19 – -0.02) |  |
| Random Effects | | | | | | | | |  |
| σ^2^ | 0.20 | | 0.20 | | | 0.18 | | |  |
| τ_00_ | 0.40 _code_ | | 0.41 _code_ | | | 0.37 _code_ | | |  |
| τ_11_ |  | |  | | | 0.00 _code.time_ | | |  |
| ρ_01_ |  | |  | | | -0.01 _code_ | | |  |
| N | 33 _code_ | | 33 _code_ | | | 33 _code_ | | |  |
| Observations | 271 | | 271 | | | 271 | | |  |
| Marginal R^2^ / Conditional R^2^ | 0.000 / 0.663 | | 0.008 / 0.678 | | | 0.009 / 0.704 | | |  |
| AIC | 436.127 | | 431.333 | | | 430.879 | | |  |
| log-Likelihood | -215.064 | | -211.667 | | | -209.439 | | |  |
| *Learning intention* | Model 1 | | Model 2 | | Model 3 | | | | |
| Predictors | Estimates | std. Beta | Estimates | std. Beta | Estimates | | std. Beta | | |
| (Intercept) | 4.20 ^***^ (-0.05) | -0.05 (-0.34 – 0.24) | 4.16 ^***^ (-0.05) | -0.05 (-0.34 – 0.24) | 4.17 ^***^ (-0.05) | | -0.05 (-0.34 – 0.24) | | |
| Time |  |  | 0.01  (0.02) | 0.02 (-0.05 – 0.10) | 0.01  (0.02) | | 0.02 (-0.09 – 0.13) | | |
| Random Effects | | | | | | | | | |
| σ^2^ | 0.24 | | 0.24 | | 0.19 | | | | |
| τ_00_ | 0.43 _code_ | | 0.42 _code_ | | 0.53 _code_ | | | | |
| τ_11_ |  | |  | | 0.01 _code.time_ | | | | |
| ρ_01_ |  | |  | | -0.45 _code_ | | | | |
| N | 33 _code_ | | 33 _code_ | | 33 _code_ | | | | |
| Observations | 268 | | 268 | | 268 | | | | |
| Marginal R^2^ / Conditional R^2^ | 0.000 / 0.639 | | 0.001 / 0.638 | | 0.000 / 0.712 | | | | |
| AIC | 474.723 | | 476.299 | | 459.827 | | | | |
| log-Likelihood | -234.361 | | -234.149 | | -223.913 | | | | |
|  | | | | | | | | | |

*Note*. Standardized beta coefficient in parentheses. Model 1 = baseline model, Model 2 = random intercept

Model with time, Model 3 = random intercept random slope model; AIC = Akaike information criterion;

logLik = log-likelihood

Table D3

Model parameters and goodness of fit for linear changes in enjoyment and anger (pre-actional)

| *Enjoyment* | Model 1 | | | Model 2 | | | | Model 3 | | | |  |
| --- | --- | --- | --- | --- | --- | --- | --- | --- | --- | --- | --- | --- |
| Predictors | Estimates | std. Beta | | Estimates | | std. Beta | | Estimates | | std. Beta | |  |
| (Intercept) | 3.55 ^***^ (-0.04) | -0.04 (-0.31 – 0.24) | | 3.53 ^***^ (-0.04) | | -0.04 (-0.31 – 0.24) | | 3.54 ^***^ (-0.05) | | -0.05 (-0.34 – 0.23) | |  |
| Time |  |  | | 0.00  (0.01) | | 0.01 (-0.07 – 0.09) | | -0.00  (-0.01) | | -0.01 (-0.14 – 0.12) | |  |
| Random Effects | | | | | | | | | | | |  |
| σ^2^ | 0.38 | | | 0.38 | | | | 0.28 | | | |  |
| τ_00_ | 0.52 _code_ | | | 0.52 _code_ | | | | 0.46 _code_ | | | |  |
| τ_11_ |  | | |  | | | | 0.01 _code.time_ | | | |  |
| ρ_01_ |  | | |  | | | | -0.28 _code_ | | | |  |
| N | 32 _code_ | | | 32 _code_ | | | | 32 _code_ | | | |  |
| Observations | 255 | | | 255 | | | | 255 | | | |  |
| Marginal R^2^ /  Conditional R^2^ | 0.000 / 0.578 | | | 0.000 / 0.578 | | | | 0.000 / 0.692 | | | |  |
| AIC | 559.439 | | | 561.353 | | | | 533.115 | | | |  |
| log-Likelihood | -276.720 | | | -276.676 | | | | -260.558 | | | |  |
|  | | | | | | | | | | | |  |
| *Anger* | Model 1 | | | | Model 2 | | | | Model 3 | | | |
| Predictors | Estimates | | std. Beta | | Estimates | | std. Beta | | Estimates | | std. Beta | |
| (Intercept) | 3.06 ^***^ (-0.06) | | -0.06 (-0.30 – 0.18) | | 3.07 ^***^ (-0.06) | | -0.06 (-0.30 – 0.18) | | 3.08 ^***^ (-0.07) | | -0.07 (-0.32 – 0.17) | |
| Time |  | |  | | -0.00  (-0.00) | | -0.00 (-0.11 – 0.10) | | -0.01  (-0.01) | | -0.01 (-0.16 – 0.13) | |
| Random Effects | | | | | | | | | | | | |
| σ^2^ | 0.64 | | | | 0.64 | | | | 0.54 | | | |
| τ_00_ | 0.35 _code_ | | | | 0.36 _code_ | | | | 0.38 _code_ | | | |
| τ_11_ |  | | | |  | | | | 0.01 _code.time_ | | | |
| ρ_01_ |  | | | |  | | | | -0.45 _code_ | | | |
| N | 31 _code_ | | | | 31 _code_ | | | | 31 _code_ | | | |
| Observations | 229 | | | | 229 | | | | 229 | | | |
| Marginal R^2^ /  Conditional R^2^ | 0.000 / 0.358 | | | | 0.000 / 0.359 | | | | 0.000 / 0.453 | | | |
| AIC | 601.586 | | | | 603.582 | | | | 595.350 | | | |
| log-Likelihood | -297.793 | | | | -297.791 | | | | -291.675 | | | |

*Note*. Standardized beta coefficient in parentheses. Model 1 = baseline model, Model 2 = random intercept

Model with time, Model 3 = random intercept random slope model; AIC = Akaike information criterion;

logLik = log-likelihood

Table D4

Model parameters and goodness of fit for linear changes in monitoring and organization

| *Monitoring* | Model 1 | | Model 2 | | Model 3 | | | |  |
| --- | --- | --- | --- | --- | --- | --- | --- | --- | --- |
| Predictors | Estimates | std. Beta | Estimates | std. Beta | Estimates | | std. Beta | |  |
| (Intercept) | 4.05 ^***^ (-0.09) | -0.09 (-0.37 – 0.19) | 4.03 ^***^ (-0.09) | -0.09 (-0.37 – 0.19) | 4.06 ^***^ (-0.10) | | -0.10 (-0.39 – 0.18) | |  |
| Time |  |  | 0.00  (0.01) | 0.01 (-0.08 – 0.09) | -0.01  (-0.02) | | -0.02 (-0.12 – 0.09) | |  |
| Random Effects | | | | | | | | |  |
| σ^2^ | 0.49 | | 0.49 | | 0.46 | | | |  |
| τ_00_ | 0.65 _code_ | | 0.65 _code_ | | 0.47 _code_ | | | |  |
| τ_11_ |  | |  | | 0.00 _code.time_ | | | |  |
| ρ_01_ |  | |  | | 0.27 _code_ | | | |  |
| N | 33 _code_ | | 33 _code_ | | 33 _code_ | | | |  |
| Observations | 253 | | 253 | | 253 | | | |  |
| Marginal R^2^ /  Conditional R^2^ | 0.000 / 0.570 | | 0.000 / 0.569 | | 0.000 / 0.610 | | | |  |
| AIC | 623.985 | | 625.942 | | 625.705 | | | |  |
| log-Likelihood | -308.993 | | -308.971 | | -306.852 | | | |  |
|  | | | | | | | | |  |
| *Organization* | Model 1 | | **Model 2** | | | Model 3 | | | |
| Predictors | Estimates | std. Beta | Estimates | std. Beta | | Estimates | | std. Beta | |
| (Intercept) | 3.94 ^***^ (-0.14) | -0.14 (-0.43 – 0.16) | 4.19 ^***^ (-0.15) | -0.15 (-0.45 – 0.15) | | 4.18 ^***^ (-0.14) | | -0.14 (-0.44 – 0.17) | |
| Time |  |  | -0.06 ^*^ (-0.11) | -0.11 (-0.19 – -0.02) | | -0.05  (-0.10) | | -0.10 (-0.23 – 0.03) | |
| Random Effects | | | | | | | | | |
| σ^2^ | 0.76 | | 0.74 | | | 0.59 | | | |
| τ_00_ | 1.14 _code_ | | 1.21 _code_ | | | 1.73 _code_ | | | |
| τ_11_ |  | |  | | | 0.02 _code.time_ | | | |
| ρ_01_ |  | |  | | | -0.54 _code_ | | | |
| N | 33 _code_ | | 33 _code_ | | | 33 _code_ | | | |
| Observations | 237 | | 237 | | | 237 | | | |
| Marginal R^2^ /  Conditional R^2^ | 0.000 / 0.600 | | 0.010 / 0.626 | | | 0.008 / 0.700 | | | |
| AIC | 693.459 | | 689.563 | | | 677.732 | | | |
| log-Likelihood | -343.730 | | -340.781 | | | -332.866 | | | |
|  | | | | | | | | | |

*Note*. Standardized beta coefficient in parentheses. Model 1 = baseline model, Model 2 = random intercept

Model with time, Model 3 = random intercept random slope model; AIC = Akaike information criterion;

logLik = log-likelihood

Table D5

Model parameters and goodness of fit for linear changes in enjoyment and anger (post-

actional)

| *Enjoyment* | Model 1 | | | Model 2 | | | | Model 3 | | | |  |
| --- | --- | --- | --- | --- | --- | --- | --- | --- | --- | --- | --- | --- |
| Predictors | Estimates | std. Beta | | Estimates | | std. Beta | | Estimates | | std. Beta | |  |
| (Intercept) | 3.53 ^***^ (-0.05) | -0.05 (-0.31 – 0.21) | | 3.50 ^***^ (-0.05) | | -0.05 (-0.31 – 0.21) | | 3.51 ^***^ (-0.06) | | -0.06 (-0.32 – 0.21) | |  |
| MZP |  |  | | 0.01  (0.02) | | 0.02 (-0.08 – 0.11) | | 0.00  (0.01) | | 0.01 (-0.11 – 0.13) | |  |
| Random Effects | | | | | | | | | | | |  |
| σ^2^ | 0.47 | | | 0.47 | | | | 0.42 | | | |  |
| τ_00_ | 0.42 _code_ | | | 0.41 _code_ | | | | 0.27 _code_ | | | |  |
| τ_11_ |  | | |  | | | | 0.01 _code.MZP_ | | | |  |
| ρ_01_ |  | | |  | | | | -0.01 _code_ | | | |  |
| N | 32 _code_ | | | 32 _code_ | | | | 32 _code_ | | | |  |
| Observations | 244 | | | 244 | | | | 244 | | | |  |
| Marginal R^2^ / Conditional R^2^ | 0.000 / 0.471 | | | 0.000 / 0.469 | | | | 0.000 / 0.530 | | | |  |
| AIC | 577.743 | | | 579.615 | | | | 573.600 | | | |  |
| log-Likelihood | -285.871 | | | -285.808 | | | | -280.800 | | | |  |
| *Anger* | Model 1 | | | | Model 2 | | | | Model 3 | | | |
| Predictors | Estimates | | std. Beta | | Estimates | | std. Beta | | Estimates | | std. Beta | |
| (Intercept) | 3.13 ^***^ (-0.05) | | -0.05 (-0.26 – 0.17) | | 3.06 ^***^ (-0.04) | | -0.04 (-0.25 – 0.17) | | 3.07 ^***^ (-0.04) | | -0.04 (-0.25 – 0.17) | |
| Time |  | |  | | 0.02  (0.04) | | 0.04 (-0.08 – 0.15) | | 0.01  (0.03) | | 0.03 (-0.09 – 0.16) | |
| Random Effects | | | | | | | | | | | | |
| σ^2^ | 0.76 | | | | 0.75 | | | | 0.74 | | | |
| τ_00_ | 0.25 _code_ | | | | 0.25 _code_ | | | | 0.12 _code_ | | | |
| τ_11_ |  | | | |  | | | | 0.00 _code.time_ | | | |
| ρ_01_ |  | | | |  | | | | 0.45 _code_ | | | |
| ICC | 0.25 | | | | 0.25 | | | | 0.27 | | | |
| N | 33 _code_ | | | | 33 _code_ | | | | 33 _code_ | | | |
| Observations | 227 | | | | 227 | | | | 227 | | | |
| Marginal R^2^ / Conditional R^2^ | 0.000 / 0.250 | | | | 0.001 / 0.251 | | | | 0.001 / 0.270 | | | |
| AIC | 624.342 | | | | 625.916 | | | | 627.501 | | | |
| log-Likelihood | -309.171 | | | | -308.958 | | | | -307.751 | | | |

*Note*. Standardized beta coefficient in parentheses. Model 1 = baseline model, Model 2 = random intercept

Model with time, Model 3 = random intercept random slope model; AIC = Akaike information criterion;

logLik = log-likelihood

Figure D1

Figure D1: Task relevance over time

Figure D2

Figure D2: Subjective competence over time
